# Trends and patterns in the global burden of intracerebral hemorrhage: a comprehensive analysis from 1990 to 2019

**DOI:** 10.3389/fneur.2023.1241158

**Published:** 2023-11-21

**Authors:** Tong Sun, Yikai Yuan, Ke Wu, Yicheng Zhou, Chao You, Junwen Guan

**Affiliations:** ^1^Department of Neurosurgery, West China Hospital, Sichuan University, Chengdu, China; ^2^Department of Neurosurgery, Xichang People’s Hospital, Xichang, Liangshan Yi Autonomous Prefecture, China

**Keywords:** intracerebral hemorrhage, disease burden, incidence, mortality, trends

## Abstract

**Objective:**

Intracerebral hemorrhage (ICH) is a significant cause of global mortality and morbidity. This study aimed to analyze the burden of ICH and its variation trends across 204 countries and territories from 1990 to 2019.

**Materials and methods:**

The study population comprised individuals of all ages and genders with ICH in 204 countries and regions between 1990 and 2019. Utilizing data from the Global Burden of Disease (GBD) study 2019, we collected information on age-standardized incidence rate (ASIR), age-standardized death rate (ASDR), and disability-adjusted life-years (DALYs) rate, which were compared by age, gender, and socio-demographic index (SDI).

**Results:**

In 2019, there were an estimated 3.4 million (3.0–3.9) incident cases, resulting in 2.8 million (2.6–3.0) deaths and 68.5 million (63.2–73.6) DALYs due to ICH. Between 1990 and 2019, the absolute number of incident cases, deaths, and DALYs increased by 43.0% (41.0–45.0), 37.0% (22.0–51.0), and 25.0% (12.0–36.0), respectively. However, the ASIR, ASDR, and DALYs showed a decreasing trend of-29.0% (−28.0 to −30.0), −36.0% (−29.0 to −43.0), and − 37.0% (−43.0 to −31.0), respectively. Countries with lower SDI tended to have higher ASIR, ASDR, and DALY rates.

**Conclusion:**

While the ASDR and DALY rates decreased from 1990 to 2019, the decline in ASIR was less pronounced. The global burden of ICH remains high, exhibiting significant variation across different genders, age groups, and SDI levels.

## Introduction

Intracerebral hemorrhage (ICH) is a significant global contributor to both mortality and morbidity, making it a major public health concern (1–3). Over the past few decades, the number of incident cases of ICH has shown a consistent upward trend (4). Despite having a lower incidence rate compared to ischemic stroke, ICH carries a higher risk of mortality and morbidity, resulting in a growing burden on healthcare systems and economies (5, 6).

The Global Burden of Diseases, Injuries, and Risk Factors Study (GBD) is a comprehensive and systematic evaluation that assesses the global burden of 354 diseases and injuries across different age groups, genders, and geographical locations. This study is updated annually, providing valuable insights into the global health landscape (7–9). By conducting a systematic and comparable analysis of the burden of ICH, considering factors such as age, sex, and geographical location, policymakers and healthcare professionals can gain evidence-based information to inform healthcare strategies and implement effective interventions (10).

The findings from the GBD study of 2016 and 2017 indicated a decline in the incidence and mortality rates of stroke; however, the overall burden of stroke remained high (11, 12). In this current study, our objective was to present the first systematic analysis of the global burden of intracerebral hemorrhage (ICH) based on the latest estimates from the GBD study of 2019. To achieve this, we collected data on various parameters, including age-standardized incidence rate (ASIR), age-standardized death rate (ASDR), years of life lost (YLLs), years lived with disability (YLDs), and disability-adjusted life-years (DALYs). These measures were then compared across different age groups, genders, and socio-demographic indexes (SDI) to gain a comprehensive understanding of the burden of ICH.

### Materials and methods

We collected data including ASIR, ASDR, YLLs, YLDs, and DALYs from 1990 to 2019 in 204 countries and regions. The data were obtained from the Institute of Health Metrics and Evaluation (IHME) website.^1^ The detailed methodology used to generate these estimates has been previously described in references (7, 8, 13–15). In summary, the GBD study of 2019 covered the period from 1 January 1990 to 31 December 2019, with data analysis completed on 1 October 2020. The overall incidence of ICH was estimated using the Bayesian meta-regression model (DisMod-MR 2.1), and the standard Cause of Death Ensemble modeling (CODEm) methods were employed to estimate overall mortality (16). In essence, CODEm represents a meticulously structured analytical instrument designed for the examination of cause of death data. It employs a diverse ensemble of modeling techniques for both rates and cause fractions, offering flexibility in covariate selection to achieve superior predictive accuracy during out-of-sample testing. DisMod-MR serves as a Bayesian meta-regression tool that facilitates the comprehensive evaluation of all accessible data pertaining to disease incidence, prevalence, remission, and mortality. This tool ensures the harmonization of epidemiological parameters, maintaining consistency throughout the analysis. DALYs are calculated as the sum of YLLs and YLDs. YLLs are determined by multiplying the count of deaths by a standard life expectancy at the age of death, which is based on the lowest observed mortality rates in any population worldwide with over 5 million individuals. YLDs are computed by multiplying the prevalence of individual disease consequences, also known as “sequelae,” by a disability weight. This weight quantifies the relative severity of a sequela on a scale ranging from 0 (representing “full health”) to 1 (representing death). Disability weights have been derived from data collected in nine population surveys as well as from an open-access internet survey. In these surveys, respondents are presented with descriptions of various health states and are asked to choose the “healthier” option from random pairs of health states. In the GBD study, age-standardized populations were computed using the GBD global age standard. The GBD analyzes conducted in 2013, 2015, and 2016 utilized age-specific proportional distributions from the United Nations Population Division’s World Population Prospects 2012 revision, covering the period from 2010 to 2035. These distributions were employed to construct a standard population age structure, employing a non-weighted mean across all national locations and years in this range. For instance, the GBD study 2017 updated the standard population age structure by taking the non-weighted mean of age-specific proportional distributions from the GBD 2017 population estimates. This update was applied to all national locations with a population exceeding 5 million people in 2017 (17). This methodology was consistently applied in GBD 2019, using the GBD 2019 population estimates (7).

Each estimate was calculated as the mean of 1,000 draws from the posterior distribution, considering factors such as age, sex, location, and year. The 95% uncertainty intervals (UI) were determined as the 25th and 975th values of the ordered draws, with statistical significance defined as a 95% UI excluding zero for all estimates.

On the basis of the GBD study, ICH was defined based on the WHO diagnosis criteria as a non-traumatic, primary event identified by brain imaging, rapidly developing clinical signs of focal (at times global) disturbance of cerebral function lasting for more than 24 h or leading to death with no apparent cause other than that of vascular origin (11, 12). Briefly, the diagnosis of ICH is typically made based on medical history, clinical evaluation, and brain imaging including computed tomography (CT) or magnetic resonance imaging (MRI). A thorough medical history, including any history of hypertension or anticoagulant medication use, is essential in establishing the cause and risk factors for ICH. The patient typically presents with acute neurological symptoms, which can include sudden severe headache, altered consciousness, weakness, numbness, or loss of function in one or more parts of the body, difficulty speaking or understanding speech, and other signs of neurological dysfunction. Diagnostic imaging, particularly CT or MRI, is crucial in confirming the diagnosis. An incident or new case of ICH was defined as the occurrence of first-ever ICH (18).

In order to examine the relationship between the development status of regions or countries and the burden of disease, a sociodemographic index (SDI) ranging from 0 to 1 was utilized, which provides a comprehensive reflection of a location’s development status. SDI is calculated as the geometric mean of normalized values for three key factors: income *per capita*, average years of schooling among the population aged 15 and over, and the total fertility rate. A higher value on the SDI scale indicates a more favorable development status. The countries were then categorized into five SDI quintiles: low, low-middle, middle, high-middle, and high. This classification allowed for the assessment of the disease burden in relation to the varying levels of development across different regions and countries.

The figures were generated through the GBD Results Tool and the GBD Compare Tool.

## Results

### Incidence

**Figure 1:**
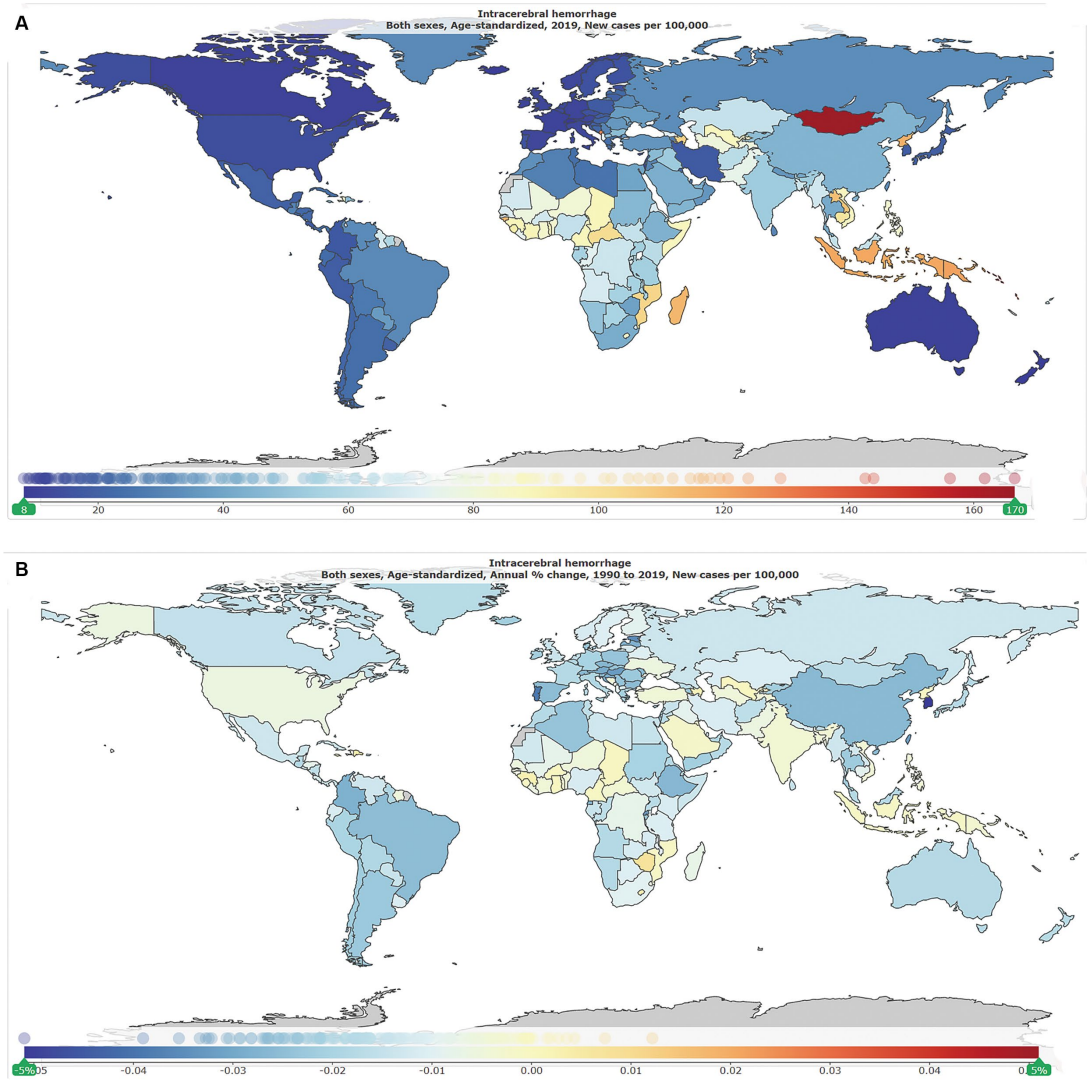
The global disease burden of intracranial hemorrhage for both genders in 204 countries and territories. **(A)** Age-standardized incidence rate per 100,000 of intracranial hemorrhage cases in 2019. **(B)** Annual percentage change in age-standardized incidence rate per 100,000 of intracranial hemorrhage cases, 1990–2019.

As shown in Table 1, globally, the number of new ICH cases was 2.3 million [95% uncertainty interval (UI): 2.0–2.7] in 1990, of whom 1.2 million (1.0–1.4) were men and 1.1 million (0.9–1.3) were women, and was 3.4 million (3.0–3.9) in 2019, of whom 1.8 million (1.5–2.1) were men and 1.5 million (1.3–1.8) were women. The global ASIR per 100,000 in 2019 is shown in Figure 1A, and the annual change (%) in ASIR from 1990 to 2019 is shown in Figure 1B. The global ASIR per 100,000 was 59.0 (51.3–68.0) in 1990 and 41.8 (36.5–47.8) in 2019. As shown in Supplementary Figure S1, from 1990 to 2019, the global number of new ICH cases has increased by 43.0% (41.0–45.0) and the global ASIR decreased by −29·0% (−28.0 to −30.0).

**Table 1:** Global incidence number and rate of ICH.

Meric	Gender	Year	Value	Upper	Lower
Number	Men	2019	1,830,930.33	2,102,660.69	1,599,645.22
Number	Women	2019	1,578,192.13	1,812,731.12	1,377,250.46
Number	Both genders	2019	3,409,122.46	3,909,193.85	2,970,474.11
Rate	Men	2019	47.18	54.18	41.22
Rate	Women	2019	40.92	47.00	35.71
Rate	Both genders	2019	44.06	50.52	38.39
Number	Men	1990	1,243,369.19	1,442,506.16	1,077,441.70
Number	Women	1990	1,137,631.51	1,307,257.08	988,020.98
Number	Both genders	1990	2,381,000.70	2,751,094.88	2,060,223.74
Rate	Men	1990	46.16	53.55	40.00
Rate	Women	1990	42.83	49.22	37.20
Rate	Both genders	1990	44.51	51.42	38.5
Number	Men	2019	1,830,930.33	2,102,660.69	1,599,645.22
Number	Women	2019	1,578,192.13	1,812,731.12	1,377,250.46
Number	Both genders	2019	3,409,122.46	3,909,193.85	2,970,474.11
Rate	Men	2019	47.18	54.18	41.22
Rate	Women	2019	40.92	47.00	35.71
Rate	Both genders	2019	44.06	50.52	38.39
Number	Men	1990	1,243,369.19	1,442,506.16	1,077,441.70
Number	Women	1990	1,137,631.51	1,307,257.08	988,020.98
Number	Both genders	1990	2,381,000.70	2,751,094.88	2,060,223.74
Rate	Men	1990	46.16	53.55	40.00
Rate	Women	1990	42.83	49.22	37.20
Rate	Both genders	1990	44.51	51.42	38.5

**Figure 2:**
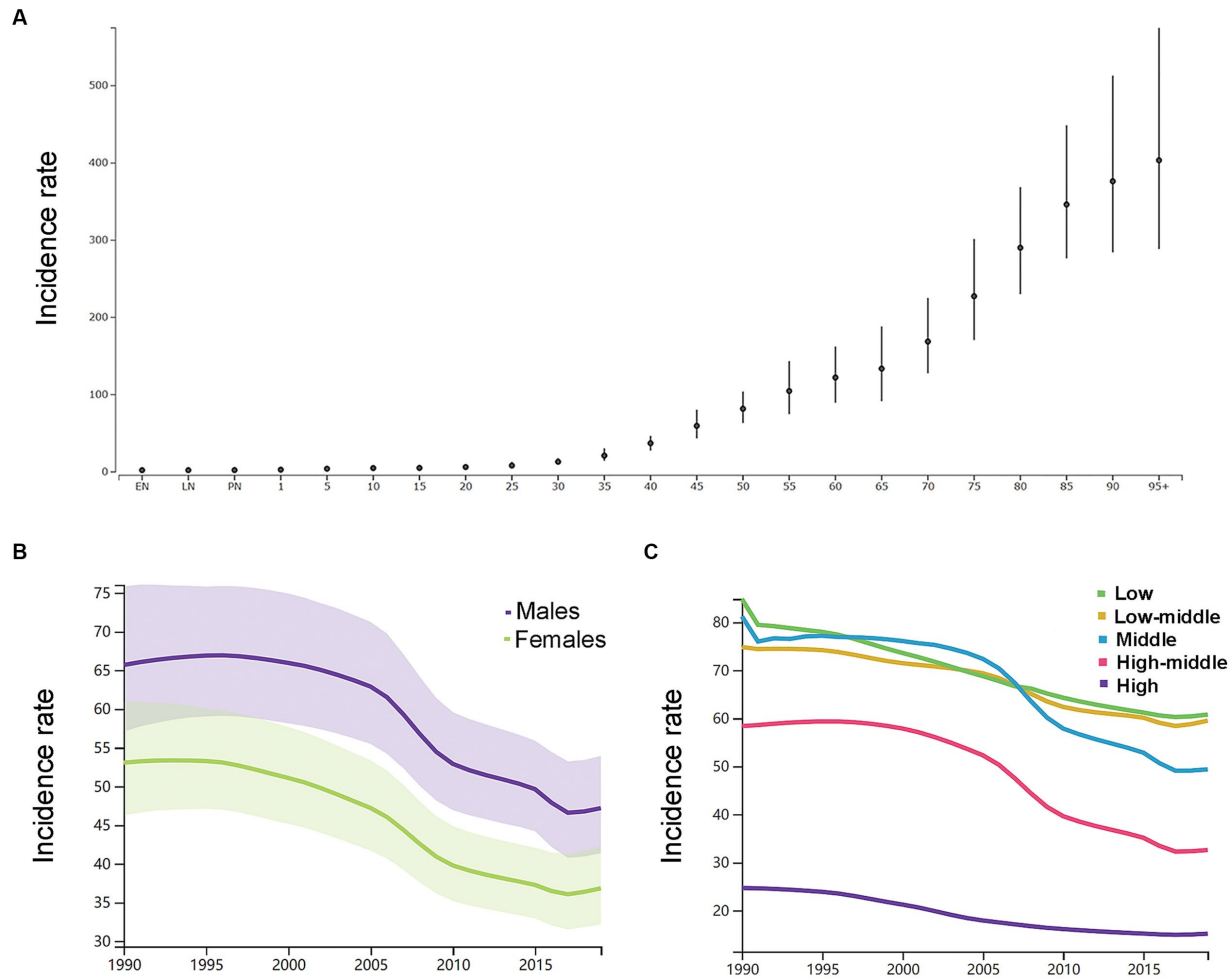
**(A)** The incidence rates per 100,000 of different age groups in 2019 in both genders. **(B)** The age-standardized incidence rates per 100,000 men and women. **(C)** The age-standardized incidence rates per 100,000 population based on 5 socio-demographic index quintiles (low, low-middle, middle, high-middle, and high). EN, early neonatal; LN, late neonatal; PN, postneonatal.

By the age group, the incidence rate per 100,000 of ICH increased with increasing age in 2019 in both genders (Figure 2A). As shown in Supplementary Table S1, in 1990, the number of new ICH cases peaked at the ages of 60–64 years in both genders (men: 158 k, 116–212; women: 120 k, 87–161), and men had a relatively larger number of new ICH cases than women at the age groups of 20–74 years. In 2019, the number of new ICH cases peaked at the ages of 55–59 years in men (223 k, 161–303) and 60–64 years in women (166 k, 121–222), and men had a relatively larger number of new ICH cases than women at the age groups of 20–74 years. Men had higher ASIR than women (Figure 2B).

As shown in Figure 2C, by SDI quintile, the ASIR in 2019 in the low SDI group, low-middle SDI group, middle SDI group, high-middle SDI group, and high SDI group was 60.8 (54.2–68.0), 59.5 (52.0–68.3), 49.4 (42.8–56.9), 32.6 (28.2–37.5), and 15.2 (13.4–17.2), respectively. From 1990 to 2019, the smallest percentage decrease in the ASIR was seen in countries in the low-middle SDI quintile (−20.0, 95% UI –22.0 to −19.0), and the greatest percentage decrease in the ASIR was seen in countries in the high-middle SDI quintile (−44.0, 95% UI –46.0 to −43.0). As shown in Supplementary Figure S2, countries in countries in Oceania (113.7, 95% UI 105.0–123.3), Southeast Asia (85.8, 95% UI 76.4–96.9), Central Asia (75.7, 95% UI 71.1–81.4), and Sub-Saharan Africa (65.5, 95% UI 58.9–73.0) had relatively high ASIR of ICH in 2019. Among all countries and territories, men in Kiribati (241.5, 223.5–261.0) and women in Mongolia (140.4, 131.0–151.2) had the highest ASIR. As shown in Supplementary Figure S2, countries in Australasia (9.6, 8.6–10.7), Western European (11.8, 10.5–13.2), high-income North America (13.3, 11.2–15.9), and high-income Asia Pacific (18.3, 16.0–21.0) had relatively low ASIR of ICH in 2019. Women (7.6, 6.5–8.6) and men (8.7, 9.9–7.7) in Switzerland had the lowest ASIR. From 1990 to 2019, the ASIR decreased the most in the Republic of Korea (total: −77.0, 95% CI, −79.0 to −75.0; women: −79, 95% CI, −82.0 to −77.0; men: −77.0, 95% CI, −79.0 to −75.0) and increased the most in Saint Vincent and the Grenadines (42.0, 95% CI, 34.0 to 50.0). In addition, the largest increase for men was in Saint Vincent and the Grenadines (34.0, 95% CI, 25.0 to 45.0), and for women was in Zimbabwe (34.0, 95% CI, 25.0 to 45.0).

### Mortality

As shown in Table 2, the number of deaths due to ICH was 2.0 million (1.9–2.3) in 1990 and 2.8 million (2.6–3.0) in 2019. The global age-standardized mortality rate per 100, 000 was 56.0 (51.5–62.2) in 1990 and was 36.0 (33.0–38.7). As shown in Supplementary Figure S3, from 1990 to 2019, the global number of deaths has increased by 37.0% (22.0–51.0) and the global age-standardized mortality rate decreased by −36.0% (−29.0 to −43.0).

**Table 2:** Global deaths number and rate of ICH.

Meric	Sex	Year	Value	Upper	Lower
Number	Men	2019	1,571,624.94	1,719,446.42	1,411,917.36
Number	Women	2019	1,314,571.46	1,451,008.99	1,169,674.38
Number	Both genders	2019	2,886,196.39	3,099,351.14	2,644,483.58
Rate	Men	2019	40.50	44.30	36.38
Rate	Women	2019	34.09	37.63	30.33
Rate	Both genders	2019	37.30	40.06	34.18
Number	Men	1990	1,078,052.20	1,207,350.51	983,323.96
Number	Women	1990	1,021,708.48	1,142,312.15	909,946.85
Number	Both genders	1990	2,099,760.68	2,328,410.73	1,932,531.32
Rate	Men	1990	40.02	44.82	36.50
Rate	Women	1990	38.47	43.01	34.26
Rate	Both genders	1990	39.25	43.52	36.12
Number	Men	2019	1,571,624.94	1,719,446.42	1,411,917.36
Number	Women	2019	1,314,571.46	1,451,008.99	1,169,674.38
Number	Both genders	2019	2,886,196.39	3,099,351.14	2,644,483.58
Rate	Men	2019	40.50	44.30	36.38
Rate	Women	2019	34.09	37.63	30.33
Rate	Both genders	2019	37.30	40.06	34.18
Number	Men	1990	1,078,052.20	1,207,350.51	983,323.96
Number	Women	1990	1,021,708.48	1,142,312.15	909,946.85
Number	Both genders	1990	2,099,760.68	2,328,410.73	1,932,531.32
Rate	Men	1990	40.02	44.82	36.50
Rate	Women	1990	38.47	43.01	34.26
Rate	Both genders	1990	39.25	43.52	36.12

**Figure 3:**
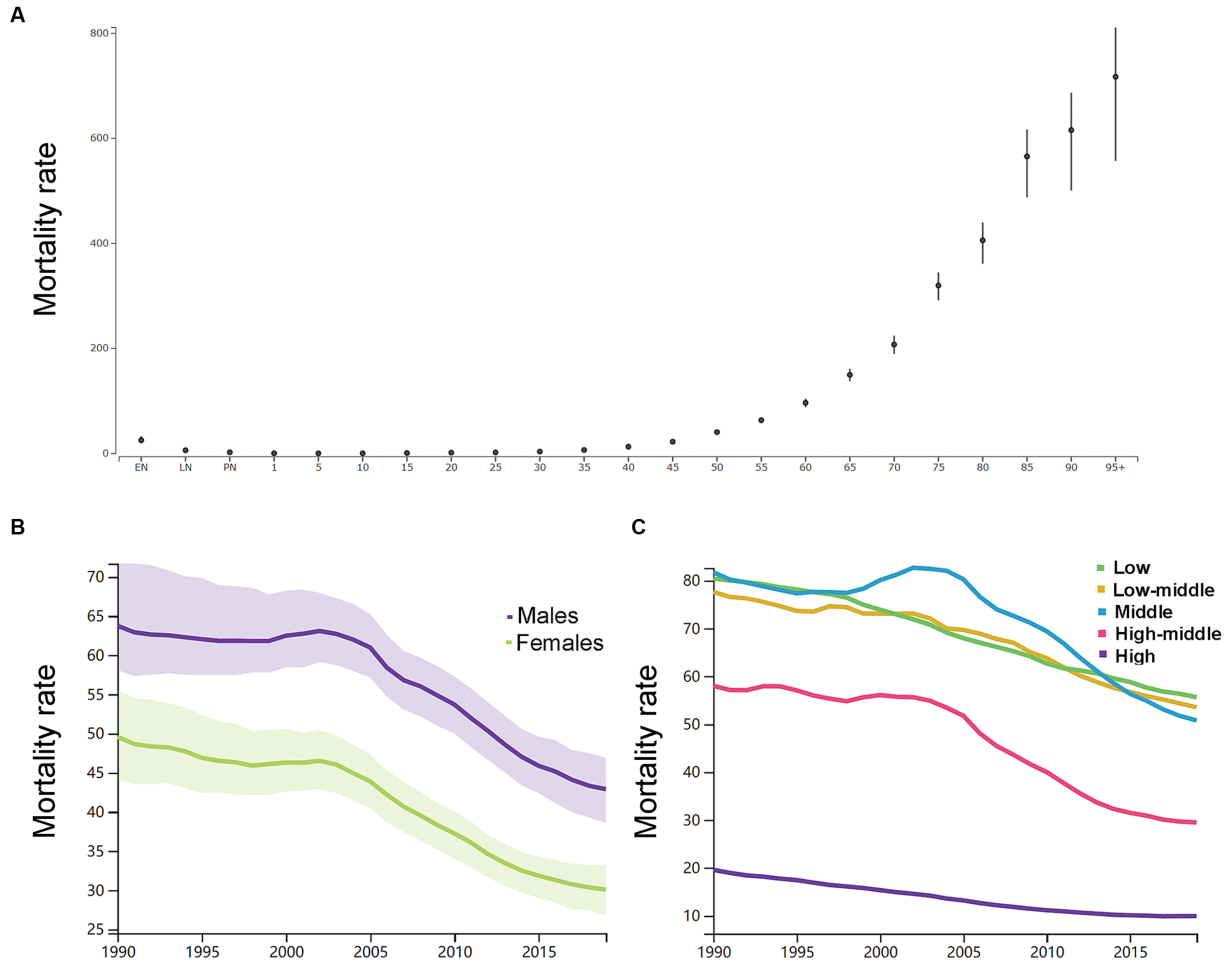
**(A)** The mortality rates per 100,000 of different age groups in 2019 in both genders. **(B)** The age-standardized mortality rates per 100,000 men and women. **(C)** The age-standardized mortality rates per 100,000 population based on 5 socio-demographic index quintiles (low, low-middle, middle, high-middle, and high). EN, early neonatal; LN, late neonatal; PN, postneonatal.

By the age group, the mortality rate per 100,000 of ICH increased with increasing age except the neonatal groups in 2019 in both genders (Figure 3A). As shown in Supplementary Table S1, in 1990, the number of deaths peaked at the ages of 65–69 years in men (160 k, 146–179) and 75–79 years in women (162 k, 144–186), and women had a relatively larger number of deaths than men at the age groups of 75 years or older. In 2019, the number of deaths peaked at the ages of 65–69 years in men (219 k, 195–241) and 75–79 years in women (192 k, 169–212), and women had a relatively larger number of deaths than men at the age groups of 75 years or older. Men had higher ASDR than women (Figure 3B).

As shown in Figure 3C, the ASDR in 2019 in the low SDI group, low-middle SDI group, middle SDI group, high-middle SDI group, and high SDI group was 55.6 (47.8–64.6), 53.5 (47.7–59.3), 50.8 (45.8–55.6), 29.5 (26.6–32.1), and 9.9 (8.9–10.6), respectively. From 1990 to 2019, the smallest percentage decrease in the ASDR was seen in countries in the low SDI quintile (−31.0, 95% UI -39.0 to −22.0), and the greatest percentage decrease in the ASDR was seen in countries in the high SDI quintile (−49.0, 95% UI -52.0 to −47.0). The region with the largest ASDR had changed from Eastern Asia (114.9, 95% UI 101.3–140.2) in 1990 to Oceania (91.5, 95% UI 69.9–118.0) in 2019. As shown in Supplementary Figure S4, in 2019, countries in Oceania (91.5, 95% UI 69.9–118.0), Central Asia (73.3, 95% UI 66.7–80.0), Southeast Asia (69.4, 95% UI 61.5–77.9), and Sub-Saharan Africa (60.6, 95% UI 52.2–69.8) had relatively high ASDR of ICH. Countries in Australasia (6.8, 95% UI 6.0–7.5), Western European (9.1, 95% UI 8.2–9.8), high-income Asia Pacific (9.9, 95% UI 8.5–10.8), and high-income North America (9.9, 95% UI 9.1–10.7) had relatively low ASDR of ICH. From 1990 to 2019, the ASIR decreased the most in the Republic of Korea (both genders: −87.0, 95% CI, −89.0 to −82.0; women: −89.0, 95% CI, −91.0 to −86.0; men: −86.0, 95% CI, −88.0 to −76.0) and increased the most in Uzbekistan (37.0, 95% CI, 15.0 to 113.0). In addition, the largest increase for men was in the Philippines (54.0, 95% UI, 2.0 to 105.0), and for women was in Azerbaijan (40.0, 95% UI, 11.0 to 75.0).

### Years of life lost, YLDs, and DALYs

As shown in Table 3, in 1990, there were 52.6 million (48.7–57.5) YLLs, 2.0 million (1.4–2.6) YLDs, and 54.7 million (50.7–59.6) DALYs due to ICH. In 2019, there were 65.3 million (60.0–70.3) YLLs, 3.2 million (2.3–4.1) YLDs, and 68.5 million (63.2–73.6) DALYs due to ICH. The global age-standardized DALYs rate per 100,000 was 1314.7 (1214.0–1433.9) in 1990 and 832.8 (769.2–894.7) in 2019. From 1990 to 2019, the global number of DALYs has increased by 25.0% (12.0–36.0), and the global age-standardized DALYs rate has decreased by −37.0% (−43.0 to −31.0).

**Table 3:** Global DALYs number and rate of ICH.

Meric	Sex	Year	Value	Upper	Lower
Number	Men	2019	39,336,302.96	42,947,067.09	35,415,117.87
Number	Women	2019	29,236,195.41	32,021,340.29	26,330,705.60
Number	Both genders	2019	68,572,498.37	73,681,972.69	63,272,310.41
Rate	Men	2019	1,013.57	1,106.61	912.53
Rate	Women	2019	758.10	830.32	682.76
Rate	Both genders	2019	886.24	952.28	817.74
Number	Men	1990	29,621,162.55	32,941,131.56	26,989,495.90
Number	Women	1990	25,105,434.67	27,503,101.24	22,771,411.01
Number	Both genders	1990	54,726,597.22	59,624,630.94	50,748,682.45
Rate	Men	1990	1,099.63	1,222.87	1,001.93
Rate	Women	1990	945.20	1,035.47	857.33
Rate	Both genders	1990	1,022.96	1,114.51	948.60
Number	Men	2019	39,336,302.96	42,947,067.09	35,415,117.87
Number	Women	2019	29,236,195.41	32,021,340.29	26,330,705.60
Number	Both genders	2019	68,572,498.37	73,681,972.69	63,272,310.41
Rate	Men	2019	1,013.57	1,106.61	912.53
Rate	Women	2019	758.10	830.32	682.76
Rate	Both genders	2019	886.24	952.28	817.74
Number	Men	1990	29,621,162.55	32,941,131.56	26,989,495.90
Number	Women	1990	25,105,434.67	27,503,101.24	22,771,411.01
Number	Both genders	1990	54,726,597.22	59,624,630.94	50,748,682.45
Rate	Men	1990	1,099.63	1,222.87	1,001.93
Rate	Women	1990	945.20	1,035.47	857.33
Rate	Both genders	1990	1,022.96	1,114.51	948.60

**Figure 4:**
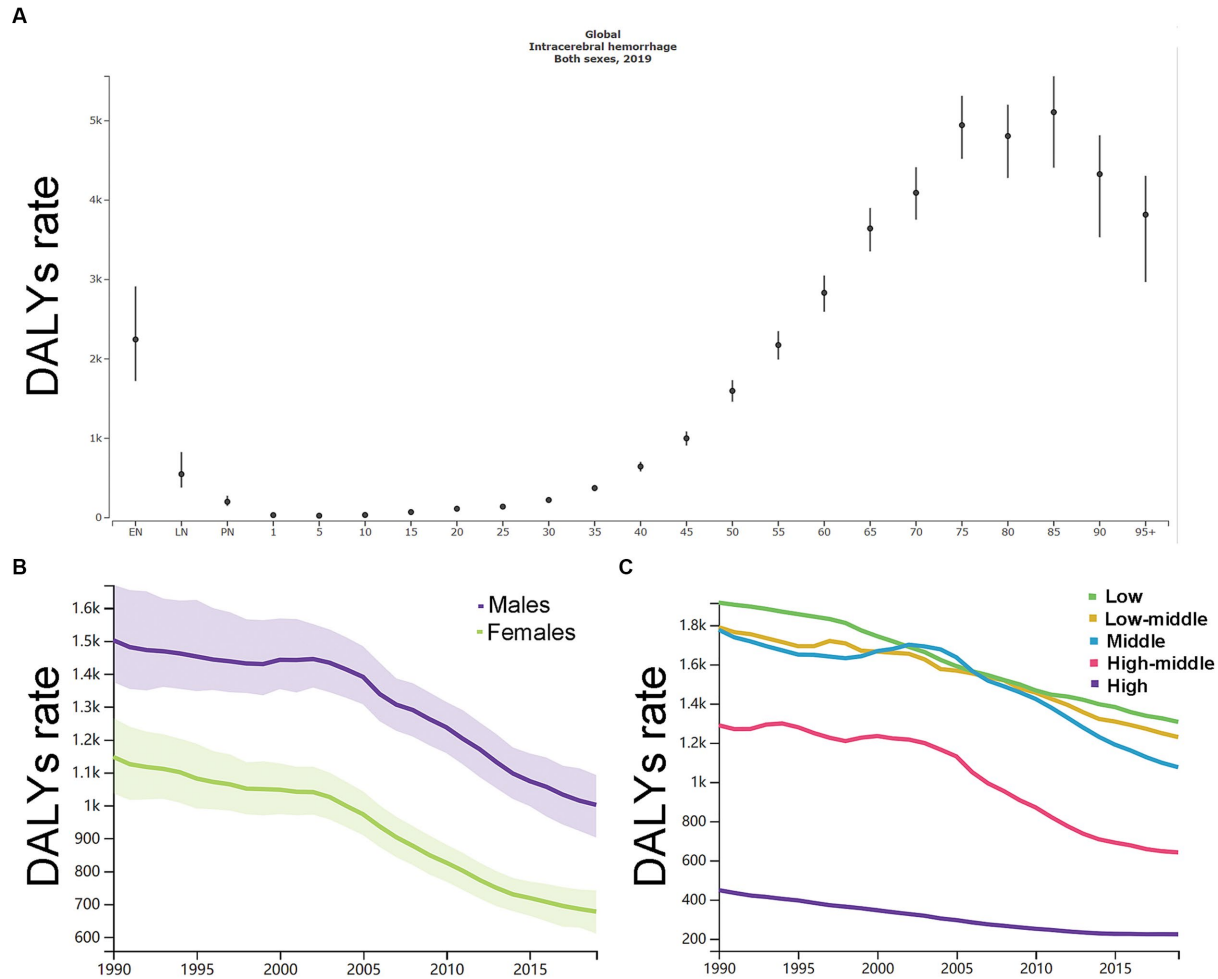
**(A)** The disability-adjusted life-years (DALYs) rates per 100,000 of different age groups. **(B)** The age-standardized DALYs rates per 100,000 men and women. **(C)** The age-standardized DALYs rates per 100,000 population based on 5 socio-demographic index quintiles (low, low-middle, middle, high-middle, and high). DALYs, disability-adjusted life-years. EN, early neonatal; LN, late neonatal; PN, postneonatal.

As shown in Supplementary Table S1, in 1990, the number of DALYs peaked at the ages of 60–64 years (7.4 million, 95% UI, 6.9 to 8.1) and the rate of DALYs peaked at the ages of 75–79 years (7721.3, 95% UI, 7099.7 to 8724.0). As shown in Figure 4A, in 2019, the number of DALYs peaked at the ages of 65–69 years (9.4 million, 95% UI, 8.7 to 10.1) and the rate of DALYs peaked at the ages of 85–89 years (5108.3, 95% UI, 4409.5 to 5560.6). Men had a higher DALY rate than women (Figure 4B).

As shown in Figure 4C, by SDI quintile, the DALYs rate in 2019 in the low SDI group, low-middle SDI group, middle SDI group, high-middle SDI group, and high SDI group was 1305.9 (1139.3–1495.5), 1228.2 (1093.2–1363.2), 1074.6 (980.2–1170.5), 640.1 (583.9–696.5), and 222.0 (208.5–236.5), respectively. From 1990 to 2019, the smallest percentage decrease in the DALYs rate was seen in countries in the low SDI quintile (−32.0, 95% UI -39.0 to −23.0) and the greatest percentage decrease in the DALYs rate was seen in countries in the high SDI quintile (−50.0, 95% UI -53.0 to −48.0). The country with the largest DALYs rate had changed from Kiribati (5612.7, 95% UI 4693.1–6652.0) in 1990 to Oceania (5250.9, 95% UI 4266.2–6444.2) in 2019 (Supplementary Figure S5).

## Discussion

In the current study, we conducted a systematic analysis of the global burden of ICH from 1990 to 2019. We compared this burden across different genders, age groups, and SDI levels. Our findings reveal that the global burden of ICH remains alarmingly high, with over 3.4 million incident cases, 2.8 million deaths, and 68.5 million DALYs reported in 2019. Notably, the global number of incident cases and deaths due to ICH has increased by approximately 43.0 and 37.0%, respectively, during the studied period. However, when considering ASIR and ASDR, most countries and regions have witnessed a decrease in these rates from 1990 to 2019. Furthermore, while the age-standardized DALYs rate, which quantifies the overall burden of ICH, has decreased, the absolute number of DALYs has actually increased from 54.7 million to 68.5 million. This increase in absolute numbers is mainly attributed to population growth and the aging of populations worldwide. These findings underscore the persistent and significant impact of ICH on global health. It highlights the need for continued efforts in prevention, early detection, and effective management of ICH, particularly in light of the increasing burden associated with population growth and aging.

Our study reveals diverse patterns and variations in the global burden of ICH across different age groups and genders. Specifically, when considering age groups, the number of incident cases of ICH peaked at the ages of 60–64 years in both genders in 1990 and at the ages of 55–59 years in men and 60–64 years in women in 2019. Between 1990 and 2019, men consistently had a higher number of new ICH cases than women in the age groups of 20–74 years. Moreover, the age-standardized incidence rate (ASIR) per 100,000 of ICH increased with advancing age, indicating a higher risk of developing ICH as individuals get older. In terms of mortality, the number of deaths due to ICH peaked at the ages of 65–69 years in men and at the ages of 75–79 years in women. Women had a relatively larger number of deaths than men in the age groups of 75 years or older. The ASDR per 100,000 of ICH generally increased with age, except in the neonatal groups. This indicates a higher mortality rate associated with ICH as individuals get older, with neonates being an exception. The age group with the highest DALYs rate, which quantifies the overall burden of ICH, changed from 75 to 79 years in 1990 to 85–89 years in 2019. This suggests that the impact of ICH on disability-adjusted life-years has shifted toward older age groups over time. Similar to our study’s conclusion, a previous systematic review and meta-analysis revealed that the incidence of ICH was observed to increase with age, and older individuals were at a higher risk of experiencing ICH compared to younger age groups (19). This phenomenon may stem from differences in lifestyle, underlying health conditions, and potential risk factors within various age brackets. It is crucial to delve into these disparities in the discussion. For instance, elderly individuals might be more susceptible to ICH due to factors such as hypertension and vascular diseases, which could be the primary drivers behind their higher incidence rates (20). The study has also highlighted variations in ICH burden between genders. A previous study found men diagnosed with ICH faced an elevated risk of both hemorrhage expansion and premature as well as delayed mortality, even after accounting for established risk factors (21). These differences could be attributed to biological dissimilarities, such as hormonal influences as well as societal and lifestyle factors. It is essential to explore these factors to gain a deeper understanding of why certain genders may be more vulnerable to ICH.

These findings emphasize the importance of considering age and sex as critical factors in understanding the burden of ICH. Considering the age-related variations in ICH burden, tailored interventions for different age groups may be necessary. For instance, public health campaigns promoting hypertension control and healthy lifestyles might be particularly beneficial for the elderly, while interventions targeting younger populations could focus on preventive measures including promoting physical activity and healthy dietary habits.

Our study findings highlight significant variations in the burden of ICH across different countries and territories. Specifically, we observed that the countries with lower SDI quintiles had the highest ASIR, ASDR, and DALYs rate associated with ICH. Additionally, the lowest percentage decrease in ASIR, ASDR, and DALYs was observed in the low or low-middle SDI quintiles. Furthermore, specific regions such as Oceania, Southeast Asia, Central Asia, and Sub-Saharan Africa exhibited relatively higher ASIR and ASDR for ICH. These regional differences in the burden of ICH are crucial for informing resource allocation strategies and the implementation of guidelines tailored to local medical conditions. Given the observed disparities, it is imperative to prioritize and allocate greater efforts toward countries in the low SDI quintile as well as developing and low-income countries. These regions often face additional challenges in terms of healthcare infrastructure, resources, and access to quality medical care. Addressing the burden of ICH in these areas will require targeted interventions, increased healthcare investments, and the development of context-specific guidelines and policies. By recognizing the regional variations in the burden of ICH, healthcare systems and policymakers can better allocate resources, implement effective interventions, and improve outcomes for individuals affected by ICH in different parts of the world (22, 23).

While previous studies have reported on the global burden of stroke using data from the Global Burden of Disease (GBD) study in 2016 and 2017, there has been a lack of separate estimates specifically focusing on ICH (11, 12). Consistent with other studies such as the Global Stroke Statistics study, GBD study 2016, and GBD study 2017, our findings also reveal a high burden associated with ICH. These findings underscore the significant impact that ICH has on global health and highlight the urgent need for targeted interventions and effective strategies to reduce the burden of this condition. By specifically analyzing the burden of ICH, our study contributes to a better understanding of the global disease landscape and provides valuable insights for policymakers, healthcare professionals, and researchers. The availability of up-to-date estimates from the GBD study 2019 allows for more accurate and comprehensive assessments of the burden of ICH, enabling more targeted approaches to prevention, treatment, and resource allocation.

### Limitations

This study has certain limitations that should be acknowledged. First, the availability of primary data in some low-middle income areas was limited within the GBD study dataset. This may have influenced the accuracy and completeness of the estimates in those regions. Second, we did not conduct a systematic analysis of the attributable risk factors for ICH, which is an important aspect that should be addressed in future studies. The GBD study of 2019 did not provide a comparison of age-standardized incidence rate (ASIR), age-standardized death rate (ASDR), and disability-adjusted life-years (DALYs) rates by race. However, we did observe significant variation in the burden of ICH across different socio-demographic index (SDI) levels and countries, indicating the complex nature of this condition and the need for further investigation.

## Conclusion

The global burden of intracerebral hemorrhage (ICH) remains substantial despite a decrease in ASIR, ASDR, and DALY rates from 1990 to 2019. However, it is important to note that the absolute number of incident cases, deaths, and DALYs associated with ICH has actually increased significantly during this period. This suggests that while progress has been made in reducing the rates of ICH, the overall impact of the condition on a global scale continues to grow.

The burden of ICH exhibits wide variations across different age groups, geographic locations, and SDI levels. It is crucial to recognize these regional differences in burden and allocate appropriate resources and interventions accordingly. Particularly, greater attention and efforts should be directed toward countries with lower SDI levels as they tend to experience higher rates of ICH and face additional challenges in terms of healthcare resources and infrastructure.

Addressing the burden of ICH requires a comprehensive approach that focuses on prevention, early detection, and effective treatment strategies. By targeting high-risk populations, implementing interventions to address modifiable risk factors, and improving access to quality healthcare services, we can work toward reducing the global burden of ICH and improving outcomes for those affected by this condition.

## Data availability statement

Publicly available datasets were analyzed in this study. This data can be found here: Institute for Health Metrics and Evaluation (IHME), Global Health Data Exchange (GHDx), 2019 Global Burden of Disease (GBD) study, http://ghdx.healthdata.org/gbd-results-tool.

## Ethics statement

Ethical review and approval was not required for the study on human participants in accordance with the local legislation and institutional requirements. Written informed consent from the patients/participants or patients/participants’ legal guardian/next of kin was not required to participate in this study in accordance with the national legislation and the institutional requirements.

## Author contributions

TS contributed to conceptualization, methodology, quality assessment, and writing the original draft. YY and YZ contributed to data curation, software, and formal analysis. YY and KW contributed to investigation. CY contributed to conceptualization, quality assessment, and manuscript revision. JG contributed to conceptualization, quality assessment, supervision, funding acquisition, and manuscript revision. All authors approved the final manuscript.

## Footnotes

^1^http://ghdx.healthdata.org/gbd-results-tool

## References

[1] Hankey GJ. Stroke. Lancet. (2017) 389:641–54. doi: 10.1016/S0140-6736(16)30962-X, PMID: 27637676

[2] Katan M Luft A. Global burden of stroke. Semin Neurol. (2018) 38:208–11. doi: 10.1055/s-0038-1649503 29791947

[3] Garg R Biller J. Recent advances in spontaneous intracerebral hemorrhage. F1000Res. (2019) 8:302. doi: 10.12688/f1000research.16357.1, PMID: 30906532 PMC6426087

[4] Ikram MA Wieberdink RG Koudstaal PJ. International epidemiology of intracerebral hemorrhage. Curr Atheroscler Rep. (2012) 14:300–6. doi: 10.1007/s11883-012-0252-1, PMID: 22538431 PMC3388250

[5] Wu S Wu B Liu M Chen Z Wang W Anderson CS. Stroke in China: advances and challenges in epidemiology, prevention, and management. Lancet Neurol. (2019) 18:394–405. doi: 10.1016/S1474-4422(18)30500-3, PMID: 30878104

[6] Feigin VL Forouzanfar MH Krishnamurthi R Mensah GA Connor M Bennett DA. Global and regional burden of stroke during 1990-2010: findings from the global burden of disease study 2010. Lancet (London, England). (2014) 383:245–55. doi: 10.1016/S0140-6736(13)61953-4, PMID: 24449944 PMC4181600

[7] Vos T Lim SS Abbafati C Abbas KM Abbasi M Abbasifard M. Global burden of 369 diseases and injuries in 204 countries and territories, 1990–2019: a systematic analysis for the global burden of disease study 2019. Lancet. (2020) 396:1204–22. doi: 10.1016/S0140-6736(20)30925-9, PMID: 33069326 PMC7567026

[8] Murray CJL Aravkin AY Zheng P Abbafati C Abbas KM Abbasi-Kangevari M. Global burden of 87 risk factors in 204 countries and territories, 1990–2019: a systematic analysis for the global burden of disease study 2019. Lancet. (2020) 396:1223–49. doi: 10.1016/S0140-6736(20)30752-2, PMID: 33069327 PMC7566194

[9] Wafa HA Wolfe CDA Emmett E Roth GA Johnson CO Wang Y. Burden of stroke in Europe: thirty-year projections of incidence, prevalence, deaths, and disability-adjusted life years. Stroke. (2020) 51:2418–27. doi: 10.1161/STROKEAHA.120.029606, PMID: 32646325 PMC7382540

[10] Hostettler IC Seiffge DJ Werring DJ. Intracerebral hemorrhage: an update on diagnosis and treatment. Expert Rev Neurother. (2019) 19:679–94. doi: 10.1080/14737175.2019.1623671 31188036

[11] Collaborators GBDS. Global, regional, and national burden of stroke, 1990-2016: a systematic analysis for the global burden of disease study 2016. Lancet Neurol. (2019) 18:439–58. doi: 10.1016/S1474-4422(19)30034-1 30871944 PMC6494974

[12] Krishnamurthi RV Ikeda T Feigin VL. Global, regional and country-specific burden of Ischaemic stroke, intracerebral Haemorrhage and subarachnoid Haemorrhage: A systematic analysis of the global burden of disease study 2017. Neuroepidemiology. (2020) 54:171–9. doi: 10.1159/000506396, PMID: 32079017

[13] Collaborators GBDD. Global age-sex-specific fertility, mortality, healthy life expectancy (HALE), and population estimates in 204 countries and territories, 1950-2019: a comprehensive demographic analysis for the global burden of disease study 2019. Lancet (London, England). (2020) 396:1160–203. doi: 10.1016/S0140-6736(20)30977-6 33069325 PMC7566045

[14] Collaborators GBDUHC. Measuring universal health coverage based on an index of effective coverage of health services in 204 countries and territories, 1990–2019: A systematic analysis for the global burden of disease study 2019. Lancet (London, England). (2020) 396:1250–84. doi: 10.1016/S0140-6736(20)30750-9 32861314 PMC7562819

[15] Smith L Shin JI Hwang SY Tizaoui K Dragioti E Jacob L. Global burden of disease study at the World Health Organization: research methods for the most comprehensive global study of disease and underlying health policies. Life Cycle. (2022) 2:e8. doi: 10.54724/lc.2022.e8

[16] Deng Y Li H Wang M Li N Tian T Wu Y. Global burden of thyroid Cancer from 1990 to 2017. JAMA Netw Open. (2020) 3:e208759. doi: 10.1001/jamanetworkopen.2020.8759, PMID: 32589231 PMC7320301

[17] Murray CJL Callender CSKH Kulikoff XR Srinivasan V Abate D Abate KH. Population and fertility by age and sex for 195 countries and territories, 1950–2017: a systematic analysis for the global burden of disease study 2017. Lancet. (2018) 392:1995–2051. doi: 10.1016/S0140-6736(18)32278-5, PMID: 30496106 PMC6227915

[18] Aho K Harmsen P Hatano S Marquardsen J Smirnov VE Strasser T. Cerebrovascular disease in the community: results of a WHO collaborative study. Bull World Health Organ. (1980) 58:113–30. PMID: 6966542 PMC2395897

[19] Van Asch CJJ Luitse MJA Rinkel GJE Van der Tweel I Algra A Klijn CJM. Incidence, case fatality, and functional outcome of intracerebral haemorrhage over time, according to age, sex, and ethnic origin: a systematic review and meta-analysis. Lancet Neurol. (2010) 9:167–76. doi: 10.1016/S1474-4422(09)70340-0, PMID: 20056489

[20] Shen J Guo F Yang P Xu F. Influence of hypertension classification on hypertensive intracerebral hemorrhage location. J Clin Hypertens. (2021) 23:1992–9. doi: 10.1111/jch.14367, PMID: 34608743 PMC8630601

[21] Marini S Morotti A Ayres AM Crawford K Kourkoulis CE Lena UK. Sex differences in intracerebral hemorrhage expansion and mortality. J Neurol Sci. (2017) 379:112–6. doi: 10.1016/j.jns.2017.05.057, PMID: 28716219 PMC5538146

[22] Qureshi AI Mendelow AD Hanley DF. Intracerebral haemorrhage. Lancet (London, England). (2009) 373:1632–44. doi: 10.1016/S0140-6736(09)60371-8, PMID: 19427958 PMC3138486

[23] Cordonnier C Demchuk A Ziai W Anderson CS. Intracerebral haemorrhage: current approaches to acute management. Lancet. (2018) 392:1257–68. doi: 10.1016/S0140-6736(18)31878-6 30319113

