# Supplementary figures and images for: Trends and patterns in the global burden of intracerebral hemorrhage: a comprehensive analysis from 1990 to 2019
Source: Front Neurol. 2023 Nov 21;14:1241158. doi: 10.3389/fneur.2023.1241158 (PMC10699537; doi:10.3389/fneur.2023.1241158)

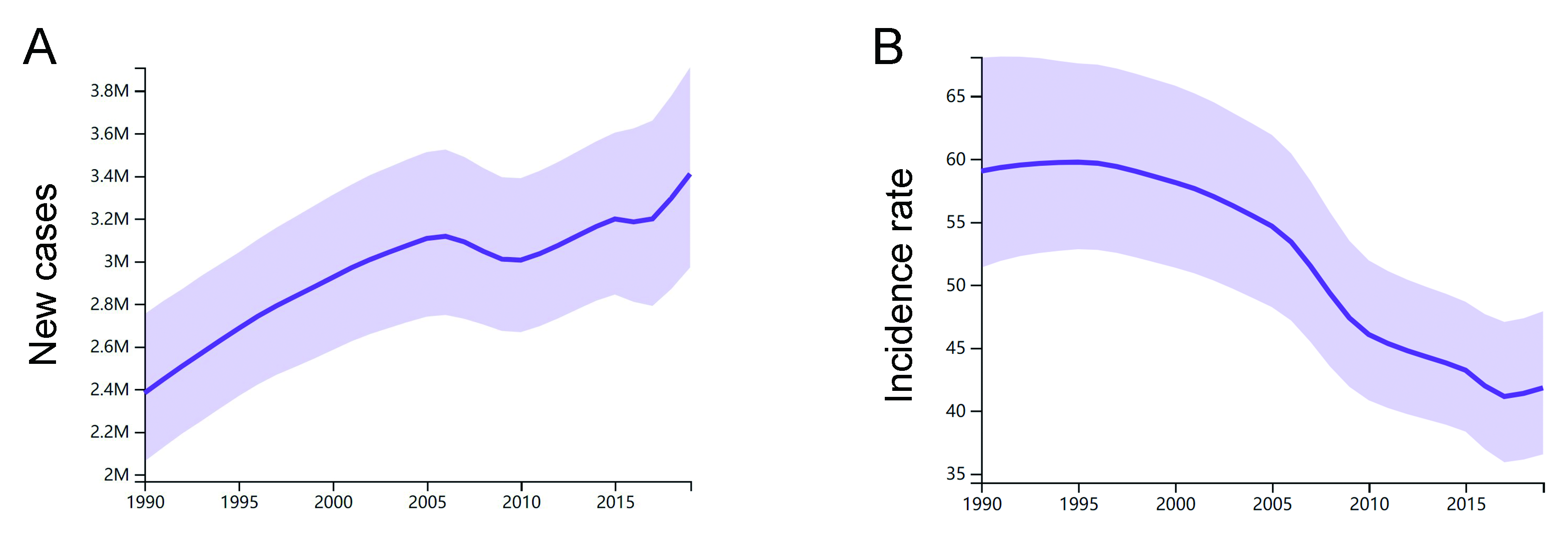

Supplement: Supplementary file 1 [file Image_1.TIF]

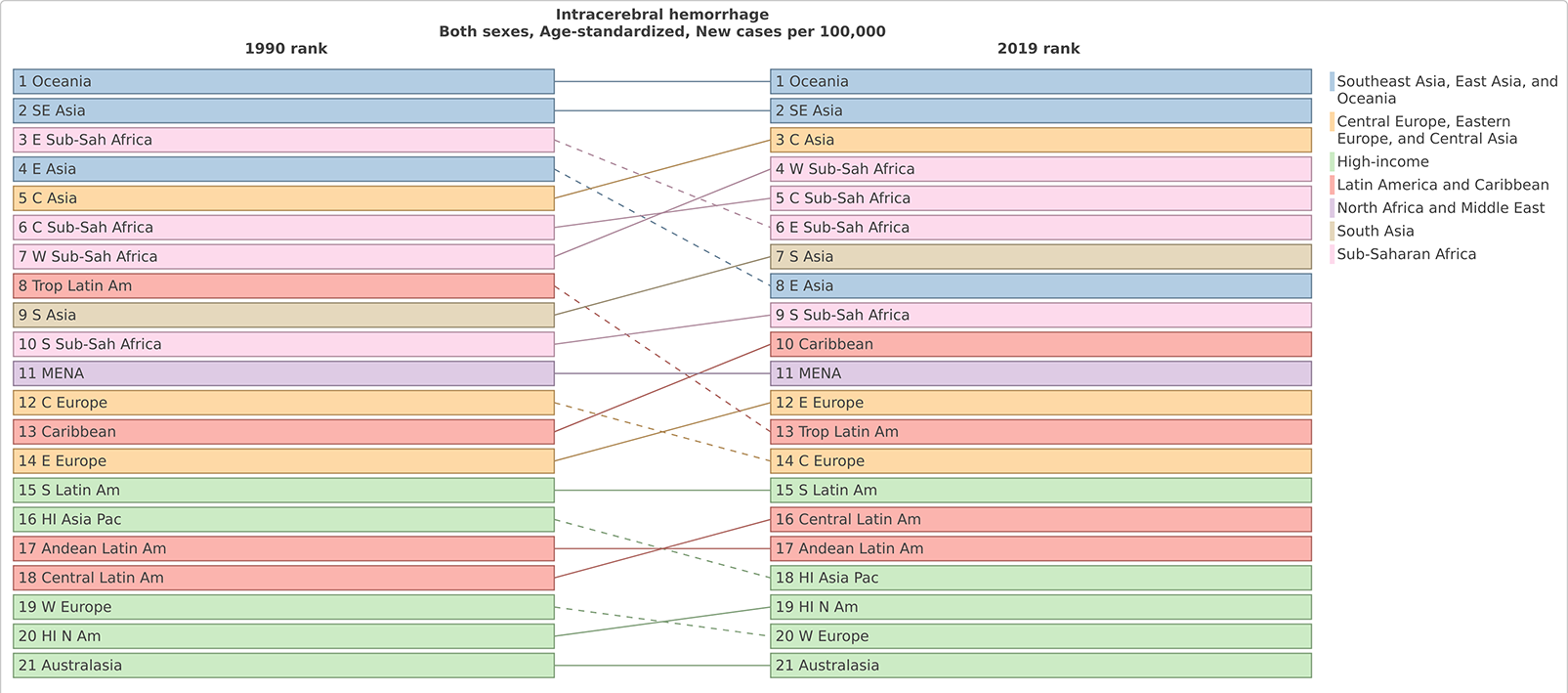

Supplement: Supplementary file 2 [file Image_2.TIF]

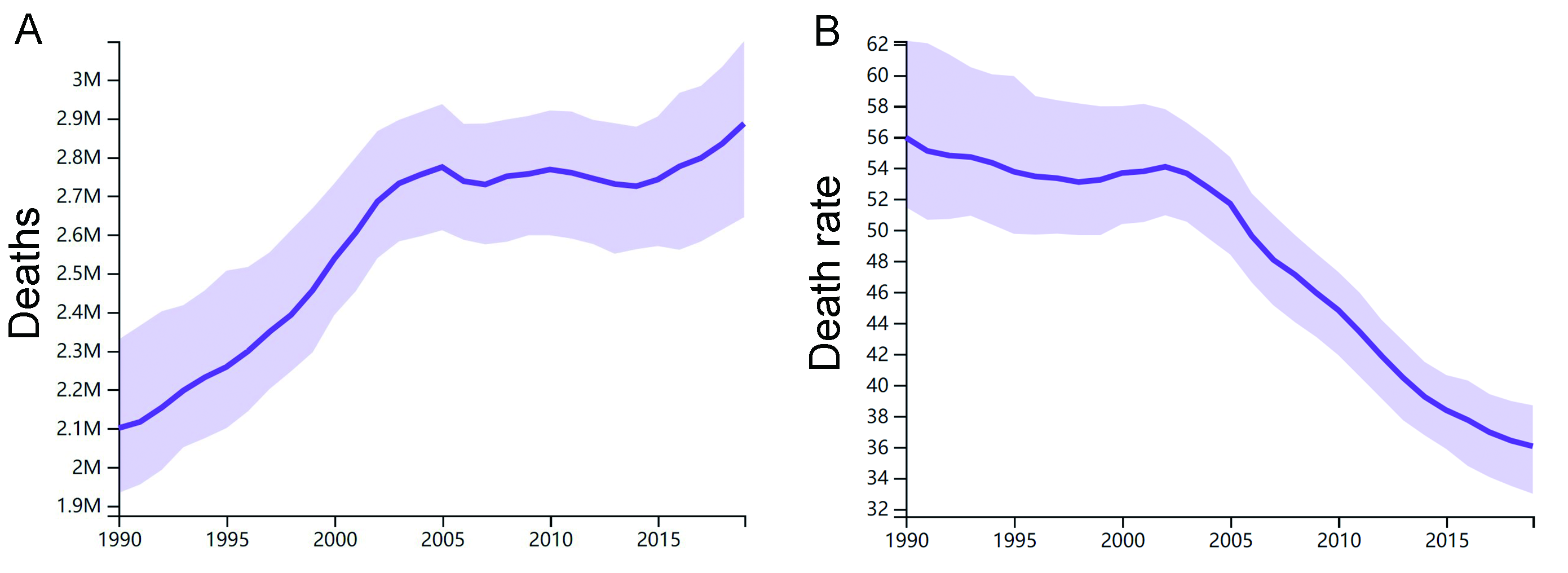

Supplement: Supplementary file 3 [file Image_3.TIF]

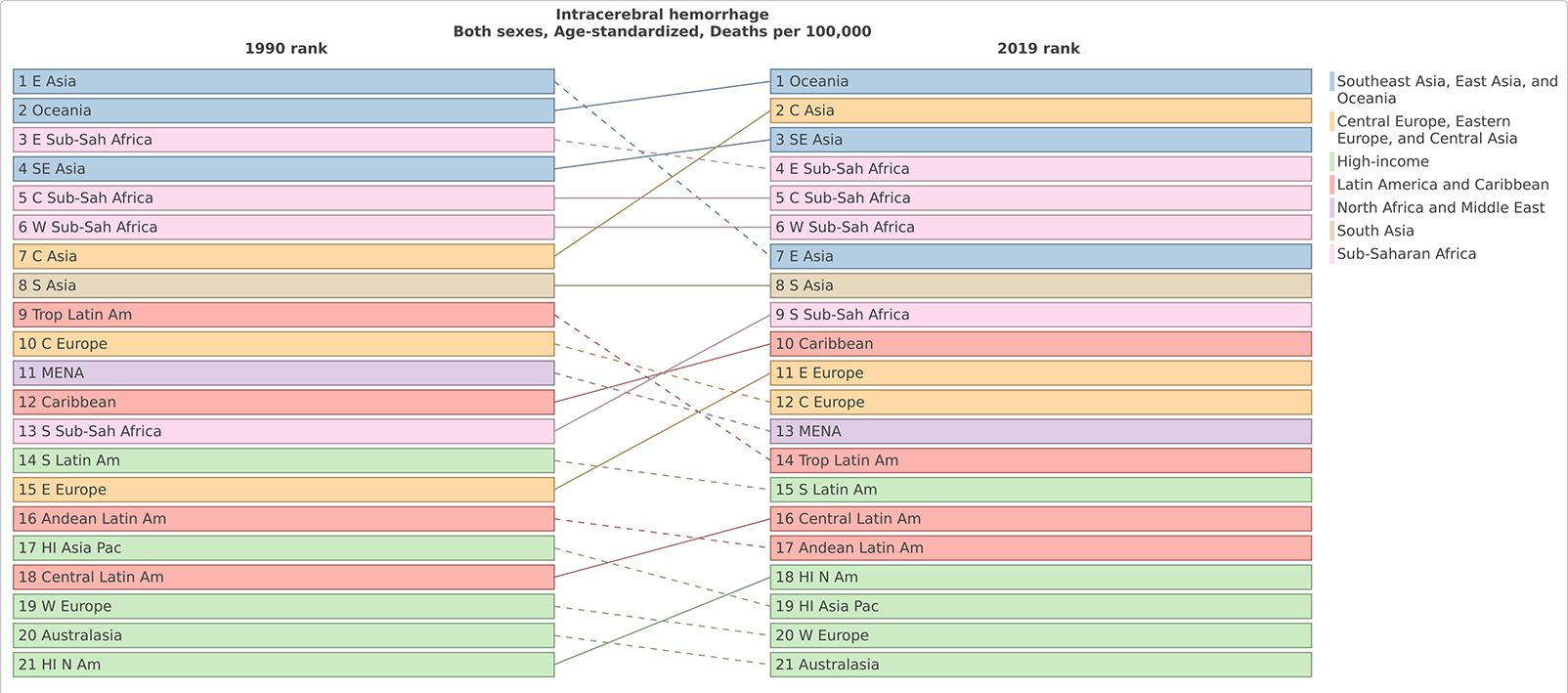

Supplement: Supplementary file 4 [file Image_4.TIF]

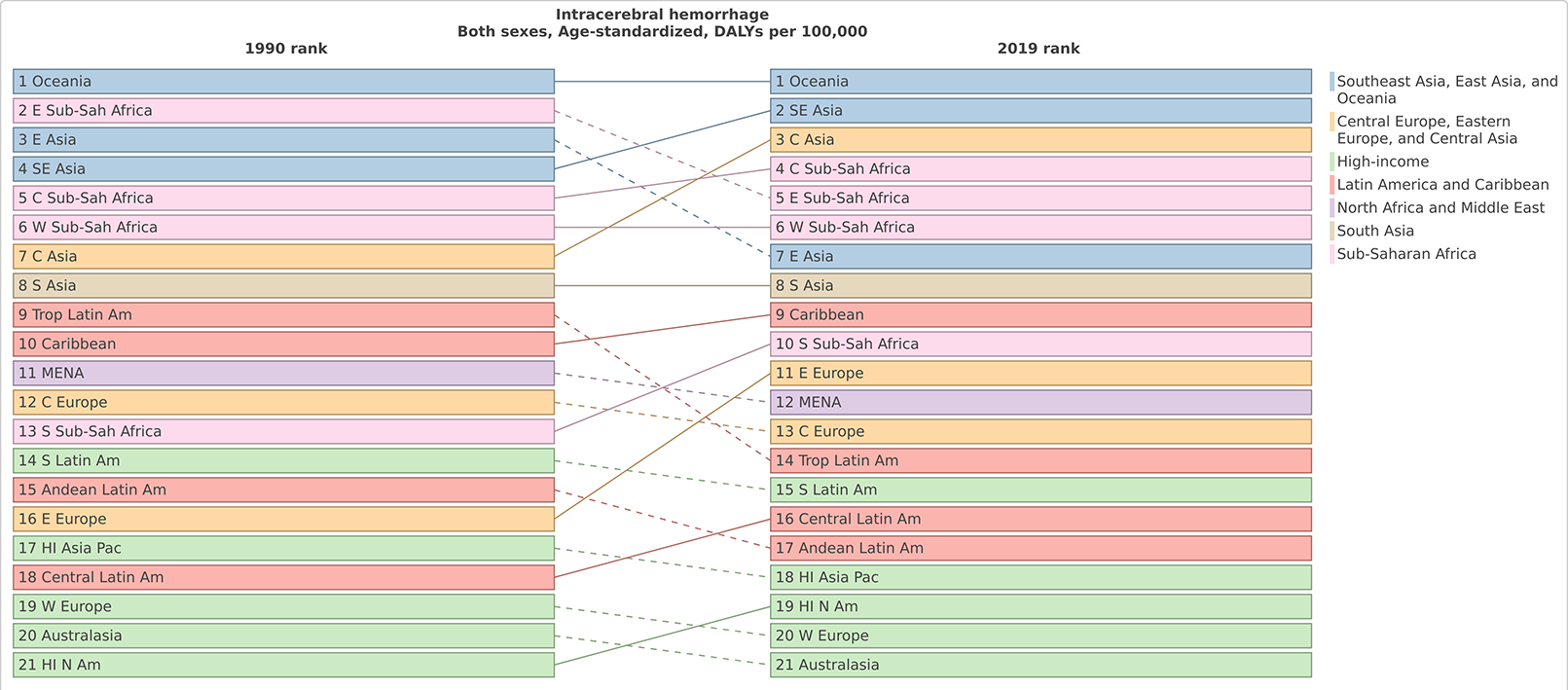

Supplement: Supplementary file 5 [file Image_5.TIF]
