# Supplementary material for: Trends and patterns in the global burden of intracerebral hemorrhage: a comprehensive analysis from 1990 to 2019
Source: Front Neurol. 2023 Nov 21;14:1241158. doi: 10.3389/fneur.2023.1241158 (PMC10699537; doi:10.3389/fneur.2023.1241158)
Supplement: Supplementary file 6 [file Table_1.DOCX]

**Supplementary Table 1. The incidence rate per 100, 000 of ICH by age groups**

| Age | Sex | Value | Upper | Lower |
| --- | --- | --- | --- | --- |
| 2019 | | | | |
| 0-6 days | Males | 1.94 | 3.60 | 0.81 |
| 0-6 days | Females | 2.42 | 4.54 | 1.04 |
| 7-27 days | Males | 1.95 | 3.58 | 0.82 |
| 7-27 days | Females | 2.43 | 4.53 | 1.05 |
| 28-364 days | Males | 2.04 | 3.59 | 0.97 |
| 28-364 days | Females | 2.56 | 4.57 | 1.25 |
| 1-4 years | Males | 2.53 | 4.25 | 1.19 |
| 1-4 years | Females | 3.21 | 5.36 | 1.53 |
| 5-9 years | Males | 3.58 | 6.01 | 1.75 |
| 5-9 years | Females | 4.60 | 7.76 | 2.25 |
| 10-14 years | Males | 4.42 | 7.40 | 2.39 |
| 10-14 years | Females | 5.45 | 9.17 | 2.96 |
| 15-19 years | Males | 4.82 | 7.84 | 2.68 |
| 15-19 years | Females | 5.45 | 9.16 | 2.93 |
| 20-24 years | Males | 6.30 | 9.31 | 4.08 |
| 20-24 years | Females | 6.25 | 9.51 | 3.75 |
| 25-29 years | Males | 8.78 | 13.50 | 5.17 |
| 25-29 years | Females | 7.59 | 12.05 | 4.36 |
| 30-34 years | Males | 15.05 | 20.14 | 10.95 |
| 30-34 years | Females | 11.09 | 15.21 | 7.71 |
| 35-39 years | Males | 24.93 | 36.07 | 17.06 |
| 35-39 years | Females | 17.07 | 24.76 | 11.60 |
| 40-44 years | Males | 43.75 | 55.14 | 32.75 |
| 40-44 years | Females | 30.32 | 38.50 | 22.56 |
| 45-49 years | Males | 70.03 | 94.18 | 50.99 |
| 45-49 years | Females | 49.01 | 66.75 | 35.55 |
| 50-54 years | Males | 96.02 | 121.98 | 74.62 |
| 50-54 years | Females | 67.55 | 85.99 | 52.23 |
| 55-59 years | Males | 122.10 | 166.08 | 88.20 |
| 55-59 years | Females | 87.79 | 121.43 | 61.98 |
| 60-64 years | Males | 141.48 | 187.18 | 104.42 |
| 60-64 years | Females | 103.90 | 138.47 | 75.63 |
| 65-69 years | Males | 153.83 | 214.97 | 106.11 |
| 65-69 years | Females | 115.20 | 165.12 | 77.98 |
| 70-74 years | Males | 189.72 | 252.18 | 143.43 |
| 70-74 years | Females | 150.06 | 200.65 | 113.62 |
| 75-79 years | Males | 250.68 | 333.60 | 187.30 |
| 75-79 years | Females | 208.29 | 276.99 | 156.93 |
| 80-84 years | Males | 317.22 | 405.96 | 250.35 |
| 80-84 years | Females | 270.92 | 343.25 | 215.36 |
| 85-89 years | Males | 369.63 | 480.93 | 295.25 |
| 85-89 years | Females | 332.25 | 430.10 | 265.86 |
| 90-94 years | Males | 379.61 | 516.24 | 289.02 |
| 90-94 years | Females | 374.85 | 512.71 | 282.28 |
| 95+ years | Males | 401.15 | 569.56 | 292.63 |
| 95+ years | Females | 404.36 | 580.36 | 287.2 |
| 0-6 days | Males | 1.94 | 3.60 | 0.81 |
| 0-6 days | Females | 2.42 | 4.54 | 1.04 |
| 7-27 days | Males | 1.95 | 3.58 | 0.82 |
| 7-27 days | Females | 2.43 | 4.53 | 1.05 |
| 28-364 days | Males | 2.04 | 3.59 | 0.97 |
| 28-364 days | Females | 2.56 | 4.57 | 1.25 |
| 1-4 years | Males | 2.53 | 4.25 | 1.19 |
| 1-4 years | Females | 3.21 | 5.36 | 1.53 |
| 5-9 years | Males | 3.58 | 6.01 | 1.75 |
| 5-9 years | Females | 4.60 | 7.76 | 2.25 |
| 10-14 years | Males | 4.42 | 7.40 | 2.39 |
| 10-14 years | Females | 5.45 | 9.17 | 2.96 |
| 15-19 years | Males | 4.82 | 7.84 | 2.68 |
| 15-19 years | Females | 5.45 | 9.16 | 2.93 |
| 20-24 years | Males | 6.30 | 9.31 | 4.08 |
| 20-24 years | Females | 6.25 | 9.51 | 3.75 |
| 25-29 years | Males | 8.78 | 13.50 | 5.17 |
| 25-29 years | Females | 7.59 | 12.05 | 4.36 |
| 30-34 years | Males | 15.05 | 20.14 | 10.95 |
| 30-34 years | Females | 11.09 | 15.21 | 7.71 |
| 35-39 years | Males | 24.93 | 36.07 | 17.06 |
| 35-39 years | Females | 17.07 | 24.76 | 11.60 |
| 40-44 years | Males | 43.75 | 55.14 | 32.75 |
| 40-44 years | Females | 30.32 | 38.50 | 22.56 |
| 45-49 years | Males | 70.03 | 94.18 | 50.99 |
| 45-49 years | Females | 49.01 | 66.75 | 35.55 |
| 50-54 years | Males | 96.02 | 121.98 | 74.62 |
| 50-54 years | Females | 67.55 | 85.99 | 52.23 |
| 55-59 years | Males | 122.10 | 166.08 | 88.20 |
| 55-59 years | Females | 87.79 | 121.43 | 61.98 |
| 60-64 years | Males | 141.48 | 187.18 | 104.42 |
| 60-64 years | Females | 103.90 | 138.47 | 75.63 |
| 65-69 years | Males | 153.83 | 214.97 | 106.11 |
| 65-69 years | Females | 115.20 | 165.12 | 77.98 |
| 70-74 years | Males | 189.72 | 252.18 | 143.43 |
| 70-74 years | Females | 150.06 | 200.65 | 113.62 |
| 75-79 years | Males | 250.68 | 333.60 | 187.30 |
| 75-79 years | Females | 208.29 | 276.99 | 156.93 |
| 80-84 years | Males | 317.22 | 405.96 | 250.35 |
| 80-84 years | Females | 270.92 | 343.25 | 215.36 |
| 85-89 years | Males | 369.63 | 480.93 | 295.25 |
| 85-89 years | Females | 332.25 | 430.10 | 265.86 |
| 90-94 years | Males | 379.61 | 516.24 | 289.02 |
| 90-94 years | Females | 374.85 | 512.71 | 282.28 |
| 95+ years | Males | 401.15 | 569.56 | 292.63 |
| 95+ years | Females | 404.36 | 580.36 | 287.2 |
| 1990 | | | | |
| 0-6 days | Males | 1.77 | 3.29 | 0.70 |
| 0-6 days | Females | 2.10 | 3.97 | 0.87 |
| 7-27 days | Males | 1.78 | 3.27 | 0.72 |
| 7-27 days | Females | 2.11 | 3.97 | 0.88 |
| 28-364 days | Males | 1.87 | 3.32 | 0.87 |
| 28-364 days | Females | 2.23 | 3.96 | 1.05 |
| 1-4 years | Males | 2.37 | 4.06 | 1.08 |
| 1-4 years | Females | 2.84 | 4.80 | 1.34 |
| 5-9 years | Males | 3.50 | 5.99 | 1.66 |
| 5-9 years | Females | 4.20 | 7.18 | 2.05 |
| 10-14 years | Males | 4.61 | 7.71 | 2.56 |
| 10-14 years | Females | 5.36 | 9.02 | 2.99 |
| 15-19 years | Males | 5.35 | 8.59 | 3.02 |
| 15-19 years | Females | 5.82 | 9.51 | 3.32 |
| 20-24 years | Males | 7.32 | 10.70 | 4.81 |
| 20-24 years | Females | 7.42 | 10.98 | 4.77 |
| 25-29 years | Males | 10.43 | 16.03 | 6.18 |
| 25-29 years | Females | 10.14 | 15.78 | 5.83 |
| 30-34 years | Males | 17.93 | 23.96 | 13.16 |
| 30-34 years | Females | 15.56 | 21.12 | 11.02 |
| 35-39 years | Males | 29.46 | 43.77 | 20.10 |
| 35-39 years | Females | 23.42 | 34.93 | 15.74 |
| 40-44 years | Males | 51.97 | 65.92 | 38.71 |
| 40-44 years | Females | 40.89 | 52.24 | 30.20 |
| 45-49 years | Males | 87.85 | 118.78 | 64.07 |
| 45-49 years | Females | 69.59 | 94.48 | 49.58 |
| 50-54 years | Males | 128.12 | 162.46 | 99.84 |
| 50-54 years | Females | 97.41 | 124.44 | 75.45 |
| 55-59 years | Males | 169.00 | 236.31 | 120.62 |
| 55-59 years | Females | 124.51 | 173.65 | 88.17 |
| 60-64 years | Males | 202.02 | 269.79 | 148.06 |
| 60-64 years | Females | 147.33 | 196.90 | 107.04 |
| 65-69 years | Males | 228.78 | 325.40 | 156.23 |
| 65-69 years | Females | 167.82 | 240.29 | 113.51 |
| 70-74 years | Males | 295.86 | 397.69 | 220.26 |
| 70-74 years | Females | 232.04 | 310.95 | 173.92 |
| 75-79 years | Males | 371.87 | 503.40 | 276.06 |
| 75-79 years | Females | 308.08 | 415.57 | 229.08 |
| 80-84 years | Males | 474.40 | 607.73 | 373.93 |
| 80-84 years | Females | 416.70 | 531.77 | 331.02 |
| 85-89 years | Males | 576.19 | 760.59 | 450.78 |
| 85-89 years | Females | 539.44 | 717.90 | 419.42 |
| 90-94 years | Males | 603.55 | 835.58 | 450.90 |
| 90-94 years | Females | 619.06 | 871.35 | 453.54 |
| 95+ years | Males | 642.99 | 933.56 | 450.41 |
| 95+ years | Females | 684.61 | 998.06 | 475.91 |
| 0-6 days | Males | 1.77 | 3.29 | 0.70 |
| 0-6 days | Females | 2.10 | 3.97 | 0.87 |
| 7-27 days | Males | 1.78 | 3.27 | 0.72 |
| 7-27 days | Females | 2.11 | 3.97 | 0.88 |
| 28-364 days | Males | 1.87 | 3.32 | 0.87 |
| 28-364 days | Females | 2.23 | 3.96 | 1.05 |
| 1-4 years | Males | 2.37 | 4.06 | 1.08 |
| 1-4 years | Females | 2.84 | 4.80 | 1.34 |
| 5-9 years | Males | 3.50 | 5.99 | 1.66 |
| 5-9 years | Females | 4.20 | 7.18 | 2.05 |
| 10-14 years | Males | 4.61 | 7.71 | 2.56 |
| 10-14 years | Females | 5.36 | 9.02 | 2.99 |
| 15-19 years | Males | 5.35 | 8.59 | 3.02 |
| 15-19 years | Females | 5.82 | 9.51 | 3.32 |
| 20-24 years | Males | 7.32 | 10.70 | 4.81 |
| 20-24 years | Females | 7.42 | 10.98 | 4.77 |
| 25-29 years | Males | 10.43 | 16.03 | 6.18 |
| 25-29 years | Females | 10.14 | 15.78 | 5.83 |
| 30-34 years | Males | 17.93 | 23.96 | 13.16 |
| 30-34 years | Females | 15.56 | 21.12 | 11.02 |
| 35-39 years | Males | 29.46 | 43.77 | 20.10 |
| 35-39 years | Females | 23.42 | 34.93 | 15.74 |
| 40-44 years | Males | 51.97 | 65.92 | 38.71 |
| 40-44 years | Females | 40.89 | 52.24 | 30.20 |
| 45-49 years | Males | 87.85 | 118.78 | 64.07 |
| 45-49 years | Females | 69.59 | 94.48 | 49.58 |
| 50-54 years | Males | 128.12 | 162.46 | 99.84 |
| 50-54 years | Females | 97.41 | 124.44 | 75.45 |
| 55-59 years | Males | 169.00 | 236.31 | 120.62 |
| 55-59 years | Females | 124.51 | 173.65 | 88.17 |
| 60-64 years | Males | 202.02 | 269.79 | 148.06 |
| 60-64 years | Females | 147.33 | 196.90 | 107.04 |
| 65-69 years | Males | 228.78 | 325.40 | 156.23 |
| 65-69 years | Females | 167.82 | 240.29 | 113.51 |
| 70-74 years | Males | 295.86 | 397.69 | 220.26 |
| 70-74 years | Females | 232.04 | 310.95 | 173.92 |
| 75-79 years | Males | 371.87 | 503.40 | 276.06 |
| 75-79 years | Females | 308.08 | 415.57 | 229.08 |
| 80-84 years | Males | 474.40 | 607.73 | 373.93 |
| 80-84 years | Females | 416.70 | 531.77 | 331.02 |
| 85-89 years | Males | 576.19 | 760.59 | 450.78 |
| 85-89 years | Females | 539.44 | 717.90 | 419.42 |
| 90-94 years | Males | 603.55 | 835.58 | 450.90 |
| 90-94 years | Females | 619.06 | 871.35 | 453.54 |
| 95+ years | Males | 642.99 | 933.56 | 450.41 |
| 95+ years | Females | 684.61 | 998.06 | 475.91 |
|  |  |  |  |  |

**Supplementary Table 2. The mortality rate per 100, 000 of ICH by age groups**

| Age | Sex | Value | Upper | Lower |
| --- | --- | --- | --- | --- |
| 2019 | | | | |
| 0-6 days | Males | 32.72 | 44.71 | 23.48 |
| 0-6 days | Females | 17.28 | 23.77 | 12.05 |
| 7-27 days | Males | 8.46 | 13.77 | 5.27 |
| 7-27 days | Females | 3.73 | 5.43 | 2.60 |
| 28-364 days | Males | 2.78 | 4.03 | 1.96 |
| 28-364 days | Females | 1.72 | 2.34 | 1.22 |
| 1-4 years | Males | 0.34 | 0.57 | 0.19 |
| 1-4 years | Females | 0.38 | 0.64 | 0.26 |
| 5-9 years | Males | 0.29 | 0.40 | 0.22 |
| 5-9 years | Females | 0.25 | 0.32 | 0.20 |
| 10-14 years | Males | 0.39 | 0.48 | 0.31 |
| 10-14 years | Females | 0.36 | 0.43 | 0.29 |
| 15-19 years | Males | 1.04 | 1.23 | 0.88 |
| 15-19 years | Females | 0.69 | 0.80 | 0.59 |
| 20-24 years | Males | 1.91 | 2.22 | 1.61 |
| 20-24 years | Females | 1.11 | 1.29 | 0.95 |
| 25-29 years | Males | 2.54 | 2.89 | 2.19 |
| 25-29 years | Females | 1.46 | 1.68 | 1.26 |
| 30-34 years | Males | 4.74 | 5.30 | 4.14 |
| 30-34 years | Females | 2.37 | 2.70 | 2.08 |
| 35-39 years | Males | 8.66 | 9.63 | 7.68 |
| 35-39 years | Females | 4.60 | 5.18 | 4.04 |
| 40-44 years | Males | 16.64 | 18.58 | 14.68 |
| 40-44 years | Females | 9.06 | 10.18 | 8.01 |
| 45-49 years | Males | 27.97 | 31.28 | 24.86 |
| 45-49 years | Females | 16.75 | 18.61 | 14.85 |
| 50-54 years | Males | 49.68 | 55.49 | 44.17 |
| 50-54 years | Females | 31.67 | 35.65 | 27.93 |
| 55-59 years | Males | 78.54 | 86.77 | 70.28 |
| 55-59 years | Females | 48.59 | 53.87 | 43.20 |
| 60-64 years | Males | 117.39 | 129.45 | 104.10 |
| 60-64 years | Females | 76.75 | 84.81 | 68.26 |
| 65-69 years | Males | 177.67 | 195.60 | 158.04 |
| 65-69 years | Females | 124.22 | 137.34 | 111.72 |
| 70-74 years | Males | 246.61 | 273.99 | 217.48 |
| 70-74 years | Females | 172.59 | 191.85 | 152.62 |
| 75-79 years | Males | 374.23 | 412.55 | 329.72 |
| 75-79 years | Females | 275.20 | 303.63 | 242.00 |
| 80-84 years | Males | 474.89 | 527.03 | 420.27 |
| 80-84 years | Females | 356.40 | 397.72 | 305.60 |
| 85-89 years | Males | 691.88 | 755.74 | 608.91 |
| 85-89 years | Females | 489.98 | 551.86 | 403.71 |
| 90-94 years | Males | 636.94 | 704.23 | 534.88 |
| 90-94 years | Females | 606.03 | 691.95 | 481.92 |
| 95+ years | Males | 624.28 | 704.68 | 498.73 |
| 95+ years | Females | 751.16 | 859.95 | 570.55 |
| 0-6 days | Males | 32.72 | 44.71 | 23.48 |
| 0-6 days | Females | 17.28 | 23.77 | 12.05 |
| 7-27 days | Males | 8.46 | 13.77 | 5.27 |
| 7-27 days | Females | 3.73 | 5.43 | 2.60 |
| 28-364 days | Males | 2.78 | 4.03 | 1.96 |
| 28-364 days | Females | 1.72 | 2.34 | 1.22 |
| 1-4 years | Males | 0.34 | 0.57 | 0.19 |
| 1-4 years | Females | 0.38 | 0.64 | 0.26 |
| 5-9 years | Males | 0.29 | 0.40 | 0.22 |
| 5-9 years | Females | 0.25 | 0.32 | 0.20 |
| 10-14 years | Males | 0.39 | 0.48 | 0.31 |
| 10-14 years | Females | 0.36 | 0.43 | 0.29 |
| 15-19 years | Males | 1.04 | 1.23 | 0.88 |
| 15-19 years | Females | 0.69 | 0.80 | 0.59 |
| 20-24 years | Males | 1.91 | 2.22 | 1.61 |
| 20-24 years | Females | 1.11 | 1.29 | 0.95 |
| 25-29 years | Males | 2.54 | 2.89 | 2.19 |
| 25-29 years | Females | 1.46 | 1.68 | 1.26 |
| 30-34 years | Males | 4.74 | 5.30 | 4.14 |
| 30-34 years | Females | 2.37 | 2.70 | 2.08 |
| 35-39 years | Males | 8.66 | 9.63 | 7.68 |
| 35-39 years | Females | 4.60 | 5.18 | 4.04 |
| 40-44 years | Males | 16.64 | 18.58 | 14.68 |
| 40-44 years | Females | 9.06 | 10.18 | 8.01 |
| 45-49 years | Males | 27.97 | 31.28 | 24.86 |
| 45-49 years | Females | 16.75 | 18.61 | 14.85 |
| 50-54 years | Males | 49.68 | 55.49 | 44.17 |
| 50-54 years | Females | 31.67 | 35.65 | 27.93 |
| 55-59 years | Males | 78.54 | 86.77 | 70.28 |
| 55-59 years | Females | 48.59 | 53.87 | 43.20 |
| 60-64 years | Males | 117.39 | 129.45 | 104.10 |
| 60-64 years | Females | 76.75 | 84.81 | 68.26 |
| 65-69 years | Males | 177.67 | 195.60 | 158.04 |
| 65-69 years | Females | 124.22 | 137.34 | 111.72 |
| 70-74 years | Males | 246.61 | 273.99 | 217.48 |
| 70-74 years | Females | 172.59 | 191.85 | 152.62 |
| 75-79 years | Males | 374.23 | 412.55 | 329.72 |
| 75-79 years | Females | 275.20 | 303.63 | 242.00 |
| 80-84 years | Males | 474.89 | 527.03 | 420.27 |
| 80-84 years | Females | 356.40 | 397.72 | 305.60 |
| 85-89 years | Males | 691.88 | 755.74 | 608.91 |
| 85-89 years | Females | 489.98 | 551.86 | 403.71 |
| 90-94 years | Males | 636.94 | 704.23 | 534.88 |
| 90-94 years | Females | 606.03 | 691.95 | 481.92 |
| 95+ years | Males | 624.28 | 704.68 | 498.73 |
| 95+ years | Females | 751.16 | 859.95 | 570.55 |
| 1990 | | | | |
| 0-6 days | Males | 75.71 | 116.27 | 46.69 |
| 0-6 days | Females | 40.58 | 58.76 | 28.98 |
| 7-27 days | Males | 31.08 | 51.61 | 15.17 |
| 7-27 days | Females | 11.52 | 16.21 | 8.43 |
| 28-364 days | Males | 10.83 | 16.94 | 6.73 |
| 28-364 days | Females | 6.89 | 10.32 | 4.38 |
| 1-4 years | Males | 1.33 | 2.37 | 0.76 |
| 1-4 years | Females | 1.35 | 2.55 | 0.81 |
| 5-9 years | Males | 0.69 | 0.89 | 0.52 |
| 5-9 years | Females | 0.59 | 0.78 | 0.46 |
| 10-14 years | Males | 0.63 | 0.77 | 0.50 |
| 10-14 years | Females | 0.58 | 0.70 | 0.47 |
| 15-19 years | Males | 1.63 | 1.94 | 1.35 |
| 15-19 years | Females | 1.21 | 1.36 | 1.05 |
| 20-24 years | Males | 2.41 | 2.84 | 1.89 |
| 20-24 years | Females | 1.83 | 2.09 | 1.57 |
| 25-29 years | Males | 3.35 | 3.84 | 2.85 |
| 25-29 years | Females | 2.74 | 3.05 | 2.45 |
| 30-34 years | Males | 5.91 | 6.73 | 5.10 |
| 30-34 years | Females | 4.31 | 4.79 | 3.82 |
| 35-39 years | Males | 11.06 | 12.66 | 9.71 |
| 35-39 years | Females | 8.14 | 9.10 | 7.25 |
| 40-44 years | Males | 21.65 | 24.84 | 18.86 |
| 40-44 years | Females | 15.52 | 17.38 | 13.77 |
| 45-49 years | Males | 39.21 | 44.45 | 34.94 |
| 45-49 years | Females | 29.71 | 33.55 | 26.27 |
| 50-54 years | Males | 75.00 | 84.77 | 67.15 |
| 50-54 years | Females | 55.53 | 61.93 | 49.49 |
| 55-59 years | Males | 121.24 | 135.65 | 108.52 |
| 55-59 years | Females | 88.42 | 98.62 | 78.09 |
| 60-64 years | Males | 183.95 | 205.20 | 167.36 |
| 60-64 years | Females | 133.17 | 149.49 | 118.31 |
| 65-69 years | Males | 280.67 | 313.04 | 255.85 |
| 65-69 years | Females | 209.71 | 235.13 | 187.31 |
| 70-74 years | Males | 397.99 | 458.46 | 357.64 |
| 70-74 years | Females | 300.34 | 346.75 | 265.30 |
| 75-79 years | Males | 576.44 | 655.31 | 526.06 |
| 75-79 years | Females | 447.37 | 515.77 | 399.11 |
| 80-84 years | Males | 654.64 | 756.87 | 587.53 |
| 80-84 years | Females | 545.07 | 639.08 | 470.06 |
| 85-89 years | Males | 851.45 | 985.25 | 756.70 |
| 85-89 years | Females | 715.84 | 834.50 | 606.85 |
| 90-94 years | Males | 891.58 | 995.13 | 766.08 |
| 90-94 years | Females | 858.58 | 995.04 | 694.16 |
| 95+ years | Males | 896.75 | 1,006.32 | 734.04 |
| 95+ years | Females | 1,004.94 | 1,161.55 | 787.43 |
| 0-6 days | Males | 75.71 | 116.27 | 46.69 |
| 0-6 days | Females | 40.58 | 58.76 | 28.98 |
| 7-27 days | Males | 31.08 | 51.61 | 15.17 |
| 7-27 days | Females | 11.52 | 16.21 | 8.43 |
| 28-364 days | Males | 10.83 | 16.94 | 6.73 |
| 28-364 days | Females | 6.89 | 10.32 | 4.38 |
| 1-4 years | Males | 1.33 | 2.37 | 0.76 |
| 1-4 years | Females | 1.35 | 2.55 | 0.81 |
| 5-9 years | Males | 0.69 | 0.89 | 0.52 |
| 5-9 years | Females | 0.59 | 0.78 | 0.46 |
| 10-14 years | Males | 0.63 | 0.77 | 0.50 |
| 10-14 years | Females | 0.58 | 0.70 | 0.47 |
| 15-19 years | Males | 1.63 | 1.94 | 1.35 |
| 15-19 years | Females | 1.21 | 1.36 | 1.05 |
| 20-24 years | Males | 2.41 | 2.84 | 1.89 |
| 20-24 years | Females | 1.83 | 2.09 | 1.57 |
| 25-29 years | Males | 3.35 | 3.84 | 2.85 |
| 25-29 years | Females | 2.74 | 3.05 | 2.45 |
| 30-34 years | Males | 5.91 | 6.73 | 5.10 |
| 30-34 years | Females | 4.31 | 4.79 | 3.82 |
| 35-39 years | Males | 11.06 | 12.66 | 9.71 |
| 35-39 years | Females | 8.14 | 9.10 | 7.25 |
| 40-44 years | Males | 21.65 | 24.84 | 18.86 |
| 40-44 years | Females | 15.52 | 17.38 | 13.77 |
| 45-49 years | Males | 39.21 | 44.45 | 34.94 |
| 45-49 years | Females | 29.71 | 33.55 | 26.27 |
| 50-54 years | Males | 75.00 | 84.77 | 67.15 |
| 50-54 years | Females | 55.53 | 61.93 | 49.49 |
| 55-59 years | Males | 121.24 | 135.65 | 108.52 |
| 55-59 years | Females | 88.42 | 98.62 | 78.09 |
| 60-64 years | Males | 183.95 | 205.20 | 167.36 |
| 60-64 years | Females | 133.17 | 149.49 | 118.31 |
| 65-69 years | Males | 280.67 | 313.04 | 255.85 |
| 65-69 years | Females | 209.71 | 235.13 | 187.31 |
| 70-74 years | Males | 397.99 | 458.46 | 357.64 |
| 70-74 years | Females | 300.34 | 346.75 | 265.30 |
| 75-79 years | Males | 576.44 | 655.31 | 526.06 |
| 75-79 years | Females | 447.37 | 515.77 | 399.11 |
| 80-84 years | Males | 654.64 | 756.87 | 587.53 |
| 80-84 years | Females | 545.07 | 639.08 | 470.06 |
| 85-89 years | Males | 851.45 | 985.25 | 756.70 |
| 85-89 years | Females | 715.84 | 834.50 | 606.85 |
| 90-94 years | Males | 891.58 | 995.13 | 766.08 |
| 90-94 years | Females | 858.58 | 995.04 | 694.16 |
| 95+ years | Males | 896.75 | 1,006.32 | 734.04 |
| 95+ years | Females | 1,004.94 | 1,161.55 | 787.43 |
|  |  |  |  |  |

**Supplementary Table 3. The DALYs rate per 100, 000 of ICH by age groups**

| Age | Sex | Value | Upper | Lower |
| --- | --- | --- | --- | --- |
| 2019 | | | | |
| 0-6 days | Males | 2,907.42 | 3,973.46 | 2,086.44 |
| 0-6 days | Females | 1,535.48 | 2,111.94 | 1,070.92 |
| 7-27 days | Males | 751.69 | 1,223.07 | 467.77 |
| 7-27 days | Females | 331.17 | 482.14 | 230.76 |
| 28-364 days | Males | 246.24 | 356.32 | 173.58 |
| 28-364 days | Females | 151.92 | 207.41 | 108.47 |
| 1-4 years | Males | 30.13 | 49.70 | 17.39 |
| 1-4 years | Females | 34.15 | 56.62 | 23.75 |
| 5-9 years | Males | 25.36 | 34.48 | 20.21 |
| 5-9 years | Females | 23.87 | 29.25 | 19.32 |
| 10-14 years | Males | 33.70 | 41.35 | 27.58 |
| 10-14 years | Females | 34.20 | 40.79 | 28.46 |
| 15-19 years | Males | 80.88 | 94.53 | 69.16 |
| 15-19 years | Females | 59.67 | 69.23 | 51.78 |
| 20-24 years | Males | 136.37 | 157.55 | 116.07 |
| 20-24 years | Females | 87.96 | 100.87 | 77.27 |
| 25-29 years | Males | 169.21 | 192.65 | 147.95 |
| 25-29 years | Females | 108.70 | 123.99 | 95.55 |
| 30-34 years | Males | 286.47 | 320.03 | 252.06 |
| 30-34 years | Females | 157.77 | 178.09 | 139.52 |
| 35-39 years | Males | 474.83 | 525.39 | 425.40 |
| 35-39 years | Females | 269.34 | 301.51 | 240.29 |
| 40-44 years | Males | 819.26 | 907.34 | 728.17 |
| 40-44 years | Females | 466.57 | 519.90 | 417.58 |
| 45-49 years | Males | 1,235.11 | 1,374.11 | 1,104.15 |
| 45-49 years | Females | 761.08 | 839.97 | 679.50 |
| 50-54 years | Males | 1,938.86 | 2,150.34 | 1,732.74 |
| 50-54 years | Females | 1,258.54 | 1,403.66 | 1,116.16 |
| 55-59 years | Males | 2,677.27 | 2,954.01 | 2,399.95 |
| 55-59 years | Females | 1,686.80 | 1,859.56 | 1,513.55 |
| 60-64 years | Males | 3,427.27 | 3,772.75 | 3,075.01 |
| 60-64 years | Females | 2,268.68 | 2,493.70 | 2,027.27 |
| 65-69 years | Males | 4,309.52 | 4,730.29 | 3,867.16 |
| 65-69 years | Females | 3,032.94 | 3,342.98 | 2,735.30 |
| 70-74 years | Males | 4,850.92 | 5,390.82 | 4,298.81 |
| 70-74 years | Females | 3,418.25 | 3,785.19 | 3,041.02 |
| 75-79 years | Males | 5,775.30 | 6,360.53 | 5,099.74 |
| 75-79 years | Females | 4,264.52 | 4,681.08 | 3,772.17 |
| 80-84 years | Males | 5,606.10 | 6,226.23 | 4,974.65 |
| 80-84 years | Females | 4,235.99 | 4,714.22 | 3,658.91 |
| 85-89 years | Males | 6,226.43 | 6,794.85 | 5,484.17 |
| 85-89 years | Females | 4,438.88 | 4,979.22 | 3,664.88 |
| 90-94 years | Males | 4,474.98 | 4,941.28 | 3,758.54 |
| 90-94 years | Females | 4,260.13 | 4,833.67 | 3,405.41 |
| 95+ years | Males | 3,317.64 | 3,726.96 | 2,662.06 |
| 95+ years | Females | 3,999.37 | 4,558.33 | 3,068.06 |
| 0-6 days | Males | 2,907.42 | 3,973.46 | 2,086.44 |
| 0-6 days | Females | 1,535.48 | 2,111.94 | 1,070.92 |
| 7-27 days | Males | 751.69 | 1,223.07 | 467.77 |
| 7-27 days | Females | 331.17 | 482.14 | 230.76 |
| 28-364 days | Males | 246.24 | 356.32 | 173.58 |
| 28-364 days | Females | 151.92 | 207.41 | 108.47 |
| 1-4 years | Males | 30.13 | 49.70 | 17.39 |
| 1-4 years | Females | 34.15 | 56.62 | 23.75 |
| 5-9 years | Males | 25.36 | 34.48 | 20.21 |
| 5-9 years | Females | 23.87 | 29.25 | 19.32 |
| 10-14 years | Males | 33.70 | 41.35 | 27.58 |
| 10-14 years | Females | 34.20 | 40.79 | 28.46 |
| 15-19 years | Males | 80.88 | 94.53 | 69.16 |
| 15-19 years | Females | 59.67 | 69.23 | 51.78 |
| 20-24 years | Males | 136.37 | 157.55 | 116.07 |
| 20-24 years | Females | 87.96 | 100.87 | 77.27 |
| 25-29 years | Males | 169.21 | 192.65 | 147.95 |
| 25-29 years | Females | 108.70 | 123.99 | 95.55 |
| 30-34 years | Males | 286.47 | 320.03 | 252.06 |
| 30-34 years | Females | 157.77 | 178.09 | 139.52 |
| 35-39 years | Males | 474.83 | 525.39 | 425.40 |
| 35-39 years | Females | 269.34 | 301.51 | 240.29 |
| 40-44 years | Males | 819.26 | 907.34 | 728.17 |
| 40-44 years | Females | 466.57 | 519.90 | 417.58 |
| 45-49 years | Males | 1,235.11 | 1,374.11 | 1,104.15 |
| 45-49 years | Females | 761.08 | 839.97 | 679.50 |
| 50-54 years | Males | 1,938.86 | 2,150.34 | 1,732.74 |
| 50-54 years | Females | 1,258.54 | 1,403.66 | 1,116.16 |
| 55-59 years | Males | 2,677.27 | 2,954.01 | 2,399.95 |
| 55-59 years | Females | 1,686.80 | 1,859.56 | 1,513.55 |
| 60-64 years | Males | 3,427.27 | 3,772.75 | 3,075.01 |
| 60-64 years | Females | 2,268.68 | 2,493.70 | 2,027.27 |
| 65-69 years | Males | 4,309.52 | 4,730.29 | 3,867.16 |
| 65-69 years | Females | 3,032.94 | 3,342.98 | 2,735.30 |
| 70-74 years | Males | 4,850.92 | 5,390.82 | 4,298.81 |
| 70-74 years | Females | 3,418.25 | 3,785.19 | 3,041.02 |
| 75-79 years | Males | 5,775.30 | 6,360.53 | 5,099.74 |
| 75-79 years | Females | 4,264.52 | 4,681.08 | 3,772.17 |
| 80-84 years | Males | 5,606.10 | 6,226.23 | 4,974.65 |
| 80-84 years | Females | 4,235.99 | 4,714.22 | 3,658.91 |
| 85-89 years | Males | 6,226.43 | 6,794.85 | 5,484.17 |
| 85-89 years | Females | 4,438.88 | 4,979.22 | 3,664.88 |
| 90-94 years | Males | 4,474.98 | 4,941.28 | 3,758.54 |
| 90-94 years | Females | 4,260.13 | 4,833.67 | 3,405.41 |
| 95+ years | Males | 3,317.64 | 3,726.96 | 2,662.06 |
| 95+ years | Females | 3,999.37 | 4,558.33 | 3,068.06 |
| 1990 | | | | |
| 0-6 days | Males | 6,728.27 | 10,332.08 | 4,149.35 |
| 0-6 days | Females | 3,605.82 | 5,221.23 | 2,575.12 |
| 7-27 days | Males | 2,760.47 | 4,584.60 | 1,347.43 |
| 7-27 days | Females | 1,022.95 | 1,440.07 | 748.78 |
| 28-364 days | Males | 957.70 | 1,498.13 | 595.52 |
| 28-364 days | Females | 609.55 | 912.21 | 387.14 |
| 1-4 years | Males | 114.86 | 204.59 | 66.26 |
| 1-4 years | Females | 116.93 | 220.25 | 70.40 |
| 5-9 years | Males | 58.32 | 75.34 | 43.96 |
| 5-9 years | Females | 51.54 | 67.15 | 40.46 |
| 10-14 years | Males | 51.80 | 62.80 | 41.51 |
| 10-14 years | Females | 50.06 | 59.81 | 41.71 |
| 15-19 years | Males | 122.86 | 145.18 | 102.39 |
| 15-19 years | Females | 95.72 | 106.69 | 84.02 |
| 20-24 years | Males | 169.44 | 198.47 | 135.93 |
| 20-24 years | Females | 135.26 | 152.55 | 117.30 |
| 25-29 years | Males | 220.09 | 250.08 | 188.87 |
| 25-29 years | Females | 187.45 | 206.89 | 169.13 |
| 30-34 years | Males | 354.29 | 400.88 | 309.48 |
| 30-34 years | Females | 269.57 | 297.36 | 241.76 |
| 35-39 years | Males | 600.31 | 686.12 | 528.16 |
| 35-39 years | Females | 455.77 | 505.40 | 410.01 |
| 40-44 years | Males | 1,056.14 | 1,208.41 | 922.34 |
| 40-44 years | Females | 775.65 | 868.75 | 692.13 |
| 45-49 years | Males | 1,712.64 | 1,929.40 | 1,531.77 |
| 45-49 years | Females | 1,319.29 | 1,479.76 | 1,172.70 |
| 50-54 years | Males | 2,895.89 | 3,261.03 | 2,599.71 |
| 50-54 years | Females | 2,171.81 | 2,417.26 | 1,936.69 |
| 55-59 years | Males | 4,093.87 | 4,578.21 | 3,687.74 |
| 55-59 years | Females | 3,019.43 | 3,371.17 | 2,679.09 |
| 60-64 years | Males | 5,323.01 | 5,937.74 | 4,866.13 |
| 60-64 years | Females | 3,888.06 | 4,333.88 | 3,472.01 |
| 65-69 years | Males | 6,765.32 | 7,500.68 | 6,186.56 |
| 65-69 years | Females | 5,078.82 | 5,692.60 | 4,547.24 |
| 70-74 years | Males | 7,798.64 | 8,973.00 | 7,032.46 |
| 70-74 years | Females | 5,905.40 | 6,794.14 | 5,232.02 |
| 75-79 years | Males | 8,891.79 | 10,063.59 | 8,125.46 |
| 75-79 years | Females | 6,912.20 | 7,930.04 | 6,181.63 |
| 80-84 years | Males | 7,755.03 | 8,933.33 | 6,965.34 |
| 80-84 years | Females | 6,477.64 | 7,593.73 | 5,621.85 |
| 85-89 years | Males | 7,691.90 | 8,874.65 | 6,837.53 |
| 85-89 years | Females | 6,494.47 | 7,569.90 | 5,527.71 |
| 90-94 years | Males | 6,273.04 | 6,997.95 | 5,406.16 |
| 90-94 years | Females | 6,051.55 | 6,981.24 | 4,924.51 |
| 95+ years | Males | 4,843.66 | 5,427.28 | 3,976.97 |
| 95+ years | Females | 5,439.85 | 6,272.18 | 4,269.15 |
| 0-6 days | Males | 6,728.27 | 10,332.08 | 4,149.35 |
| 0-6 days | Females | 3,605.82 | 5,221.23 | 2,575.12 |
| 7-27 days | Males | 2,760.47 | 4,584.60 | 1,347.43 |
| 7-27 days | Females | 1,022.95 | 1,440.07 | 748.78 |
| 28-364 days | Males | 957.70 | 1,498.13 | 595.52 |
| 28-364 days | Females | 609.55 | 912.21 | 387.14 |
| 1-4 years | Males | 114.86 | 204.59 | 66.26 |
| 1-4 years | Females | 116.93 | 220.25 | 70.40 |
| 5-9 years | Males | 58.32 | 75.34 | 43.96 |
| 5-9 years | Females | 51.54 | 67.15 | 40.46 |
| 10-14 years | Males | 51.80 | 62.80 | 41.51 |
| 10-14 years | Females | 50.06 | 59.81 | 41.71 |
| 15-19 years | Males | 122.86 | 145.18 | 102.39 |
| 15-19 years | Females | 95.72 | 106.69 | 84.02 |
| 20-24 years | Males | 169.44 | 198.47 | 135.93 |
| 20-24 years | Females | 135.26 | 152.55 | 117.30 |
| 25-29 years | Males | 220.09 | 250.08 | 188.87 |
| 25-29 years | Females | 187.45 | 206.89 | 169.13 |
| 30-34 years | Males | 354.29 | 400.88 | 309.48 |
| 30-34 years | Females | 269.57 | 297.36 | 241.76 |
| 35-39 years | Males | 600.31 | 686.12 | 528.16 |
| 35-39 years | Females | 455.77 | 505.40 | 410.01 |
| 40-44 years | Males | 1,056.14 | 1,208.41 | 922.34 |
| 40-44 years | Females | 775.65 | 868.75 | 692.13 |
| 45-49 years | Males | 1,712.64 | 1,929.40 | 1,531.77 |
| 45-49 years | Females | 1,319.29 | 1,479.76 | 1,172.70 |
| 50-54 years | Males | 2,895.89 | 3,261.03 | 2,599.71 |
| 50-54 years | Females | 2,171.81 | 2,417.26 | 1,936.69 |
| 55-59 years | Males | 4,093.87 | 4,578.21 | 3,687.74 |
| 55-59 years | Females | 3,019.43 | 3,371.17 | 2,679.09 |
| 60-64 years | Males | 5,323.01 | 5,937.74 | 4,866.13 |
| 60-64 years | Females | 3,888.06 | 4,333.88 | 3,472.01 |
| 65-69 years | Males | 6,765.32 | 7,500.68 | 6,186.56 |
| 65-69 years | Females | 5,078.82 | 5,692.60 | 4,547.24 |
| 70-74 years | Males | 7,798.64 | 8,973.00 | 7,032.46 |
| 70-74 years | Females | 5,905.40 | 6,794.14 | 5,232.02 |
| 75-79 years | Males | 8,891.79 | 10,063.59 | 8,125.46 |
| 75-79 years | Females | 6,912.20 | 7,930.04 | 6,181.63 |
| 80-84 years | Males | 7,755.03 | 8,933.33 | 6,965.34 |
| 80-84 years | Females | 6,477.64 | 7,593.73 | 5,621.85 |
| 85-89 years | Males | 7,691.90 | 8,874.65 | 6,837.53 |
| 85-89 years | Females | 6,494.47 | 7,569.90 | 5,527.71 |
| 90-94 years | Males | 6,273.04 | 6,997.95 | 5,406.16 |
| 90-94 years | Females | 6,051.55 | 6,981.24 | 4,924.51 |
| 95+ years | Males | 4,843.66 | 5,427.28 | 3,976.97 |
| 95+ years | Females | 5,439.85 | 6,272.18 | 4,269.15 |
